# Characterization and regulation of salt upregulated cyclophilin from a halotolerant strain of *Penicillium oxalicum*

**DOI:** 10.1038/s41598-023-44606-5

**Published:** 2023-10-13

**Authors:** Mangaljeet Singh, Harpreet Singh, Kirandeep Kaur, Shubhankar Shubhankar, Supreet Singh, Amarjeet Kaur, Prabhjeet Singh

**Affiliations:** 1 Department of Biotechnology, Guru Nanak Dev University, Amritsar, Punjab 143005 India; 2 Department of Bioinformatics, Hans Raj Mahila Maha Vidyalaya, Jalandhar, Punjab 144008 India; 3 Department of Microbiology, Guru Nanak Dev University, Amritsar, Punjab 143005 India

**Keywords:** Biotechnology, Computational biology and bioinformatics, Molecular biology, Structural biology

## Abstract

*Penicillium* species are an industrially important group of fungi. Cyclophilins are ubiquitous proteins and several members of this family exhibit peptidyl-prolyl *cis–trans* isomerase (PPIase) activity. We had earlier demonstrated that the salt-induced PPIase activity in a halotolerant strain of *P. oxalicum* was associated with enhanced expression of a cyclophilin gene, *PoxCYP18*. Cloning and characterization of PoxCYP18 revealed that its cDNA consists of 522 bp encoding a protein of 173 amino acid residues, with predicted molecular mass and pI values of 18.91 kDa and 8.87, respectively. The recombinant PoxCYP18 can catalyze *cis–trans* isomerization of peptidyl-prolyl bond with a catalytic efficiency of 1.46 × 10^7^ M^−1^ s^−1^ and is inhibited specifically only by cyclosporin A, with an inhibition constant of 5.04 ± 1.13 nM. PoxCYP18 consists of two cysteine residues at positions − 45 and − 170, and loses its activity under oxidizing conditions. Substitution of these residues alone or together by site-directed mutagenesis revealed that the PPIase activity of PoxCYP18 is regulated through a redox mechanism involving the formation of disulfide linkages. Heterologous expression of PoxCYP18 conferred enhanced tolerance to salt stress in transgenic *E. coli* cells, implying that this protein imparts protection to cellular processes against salt-induced damage.

## Introduction

Protein peptide bonds can exist in either *cis* or *trans* conformation, favoring the latter due to thermodynamic and geometrical considerations^1^. However, due to the cyclic five-membered ring structure of proline, 10–15% of the peptidyl-prolyl bonds have the propensity for assuming the *cis* configuration^2^. The *cis* configuration of the peptidyl-prolyl bonds introduces bends in the proteins and decreases their stability. Conversion of the *cis* peptidyl-prolyl bonds to *trans* conformation, a rate-limiting step, is therefore imperative for the proper folding of proteins. The *cis–trans* transition of peptidyl-prolyl bonds can be catalyzed only by the enzymes peptidyl-prolyl *cis–trans* isomerases (PPIases)^3^. Akin to typical enzymes, the PPIases follow the Michaelis–Menten kinetics and do not require energy for *cis–trans* isomerization activity^4,5^. Until now, four different classes of PPIases, viz. cyclophilins, FK506-binding proteins (FKBPs), parvulins and protein phosphatase 2A phosphatase activators (PTPAs), have been reported^6^. While cyclophilins and FKBPs bind cyclosporin A (CsA) and FK506/rapamycin, respectively, the parvulins show interaction with juglone (5-hydroxy-1, 4-naphthoquinone)^7–9^. Since CsA and FK506 are immunosuppressants^10^, the cyclophilins and FKBPs are together also termed as immunophilins^11^. Cyclophilins are ubiquitously present proteins and are observed in a broad range of organisms, including viruses, bacteria, fungi, mammals and plants^6,12,13^. These proteins are characterized by the presence of a cyclophilin-like domain (CLD), and are encoded by large gene families, with the number ranging between eight in *Saccharomyces cerevisiae* to 19 in humans, 83 in *Triticum aestivum* and 91 in *Brassica napus*^14–18^. Besides acting as receptors for CsA and subsequent regulators of an immune response, cyclophilins have been demonstrated to play important roles in various other cellular processes, viz. RNA processing, plant growth and development, abiotic stress adaptation, etc.^6,16^. PPIases have also been cloned and characterized from several fungi^19,20^ and are implicated in diverse functions viz., virulence^21–23^, growth and development^24^, folding of proteins^25^ and abiotic stress response^26,27^.

Recent studies in our lab resulted in the identification of 7–11 cyclophilins, 2–5 FKBPs, and 1–2 parvulins and PTPA across different species of *Penicillium*^28^. We further observed PPIase activity in the mycelia of a halotolerant strain of *P. oxalicum*, the genome of which encodes ten cyclophilins, four FKBPs and two each of parvulins and PTPAs, was enhanced significantly under salt stress (15% NaCl)^28^. The stress-induced increase in PPIase activity in *P. oxalicum* was also associated with increased expression of a cyclophilin gene, *PoxCYP18*, suggesting its role in stress response. Since information about the role of cyclophilins is lacking entirely in *Penicillium*, in the present study, we carried out cloning of cDNA encoding PoxCYP18 and characterized its biochemical properties and regulatory mechanisms to gain insight into its likely role in *P. oxalicum.*

## Results

### Bioinformatics analysis

Previous studies in our lab demonstrated that the salt-induced PPIase activity in the mycelia of a halotolerant strain of *P. oxalicum* was accompanied by enhanced expression of the cyclophilin gene *PoxCYP18*^28^. To further understand the role of this cyclophilin, we carried out cloning of its cDNA, followed by a heterologous expression, purification and characterization of the protein. In silico analysis revealed that the open reading frame (ORF) of *PoxCYP18* consists of 522 nucleotides encoding a protein of 173 amino acid (AA) residues, with predicted molecular mass and pI values of 18.91 kDa and 8.87, respectively. Comparative in silico analysis of *PoxCYP18* cDNA with the genome sequence of *P. oxalicum* revealed that the full-length gene comprises 1001 bps and contains three introns of 260, 118 and 101 bps, and four different exons of 58, 170, 107 and 187 bps. This genomic organization of the *PoxCYP18* gene was also supported by the size of PCR amplicon obtained with gene-specific primers using genomic DNA (1001 bp) and cDNA (522 bp) of *P. oxalicum* as templates (Supplementary table 1; Supplementary Fig. S1a). Computational analysis further revealed that the CLD in PoxCYP18 ranges from 16 to 172 AAs, consistent with the length in other orthologues^28^. PoxCYP18 contains two cysteine residues at AA positions 45 and 170. The nine active site residues (Arg60, Phe65, Met66, Gln68, Ala106, Phe118, Trp126, Leu127 and His131), required for CsA binding and PPIase activity, are conserved in PoxCYP18 (Supplementary Fig. S2). Analysis with the software Peptide Property Calculator indicated that compared to acidic (9.83%) and basic amino acid residues (13.29%), the neutral (39.88%) and hydrophobic residues (36.99%) are present in greater proportion. PoxCYP18 contains 20 positively (Arg + Lys) and 17 negatively (Asp + Glu) charged residues. The whole protein contains about 17.34% alpha helix, which might impart its structure a greater stability (Supplementary Fig. S1d). Phylogenetic analysis of PoxCYP18 sequence with other fungal cyclophilins clustered these proteins in a single clade (Supplementary Fig. S1c), depicting maximum similarity (90.2%) with *Aspergillus niger* orthologue, CYPA (Supplementary Fig. S1b, Supplementary table 2).

### Cloning of *PoxCYP18* cDNA and biochemical characterization of the protein

**Figure 1:**
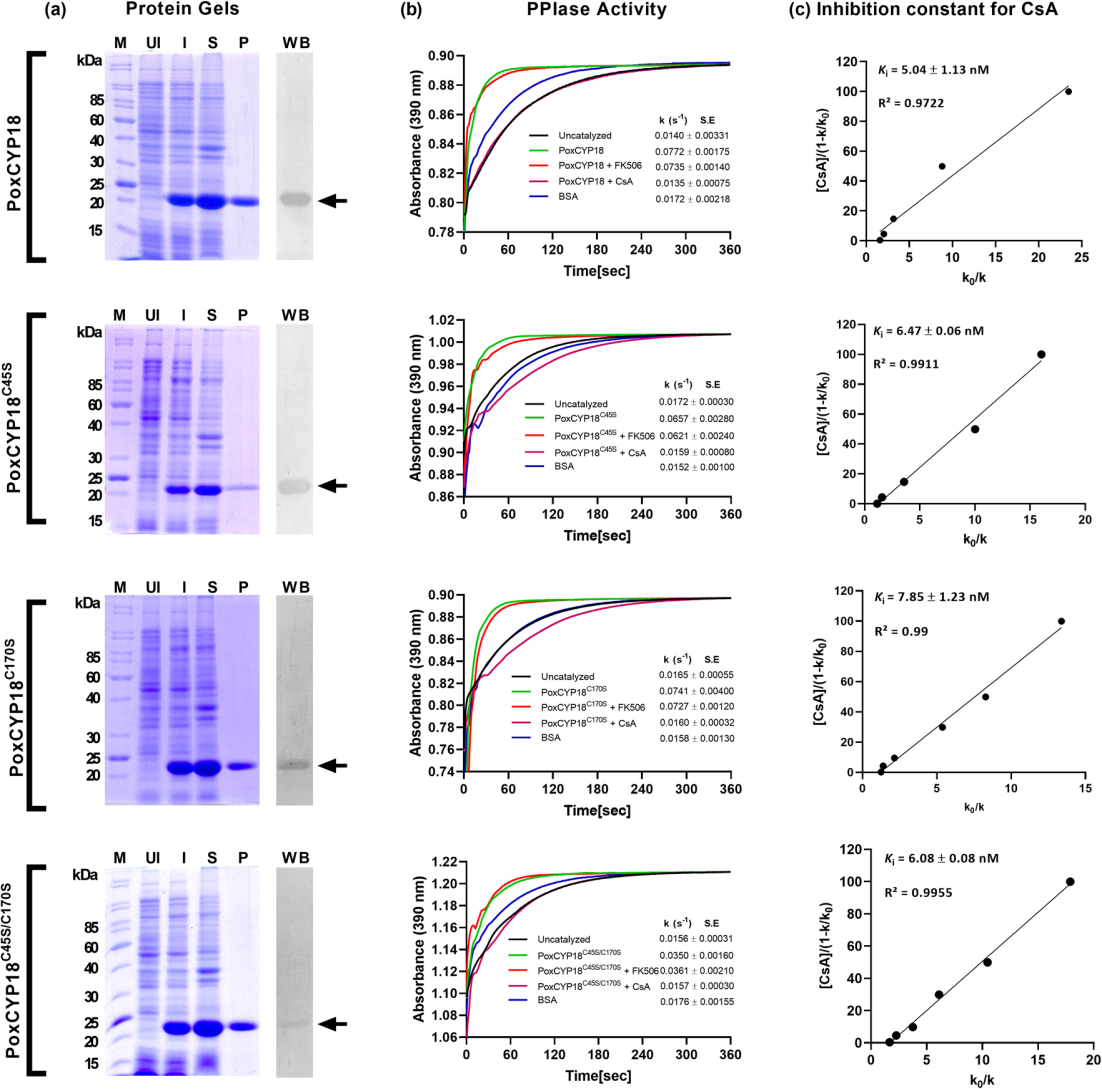
(**a**) SDS-PAGE (12%) analysis of the purified PoxCYP18 and its mutant proteins PoxCYP18^C45S^, PoxCYP18^C170S^ and PoxCYP18^C45S/C170S^ under reducing conditions. The total proteins extracted from the recombinant *E. coli* BL21(DE3)pLysS cells before (UI) and after induction with IPTG were analyzed for solubility by electrophoretic analysis of the insoluble pellet (lane IS) and soluble fractions (lane S). The recombinant proteins were purified from soluble fraction by Ni-NTA affinity column (lane P) and confirmed by immunoblotting with anti-His antibody (lane WB; the full-sized blots are shown in Supplementary Fig. 6; M: Marker). (**b**) Estimation of peptidyl prolyl cis*–trans* isomerase (PPIase) activity**.** Hydrolysis of *N*-succinyl-ala-ala-pro-phe-*p*-nitroanilidine was carried out in the presence of purified protein (4.4 µM). The rate of reaction is expressed as first-order rate constant (k). Bovine serum albumin (BSA) was used as a negative control. (**c**) Effect of cyclosporin A (CsA) on PPIase activity of the purified proteins. Inhibition constants (*K*_i_ of CsA for the native and mutants of PoxCYP18 were determined as gradient of the line of the best fit from a plot of [CsA]/(1-*k*/*k*_0_) against *k*_0_/*k*, where* k* is the rate constant at any given CsA concentration and ko is the rate constant in the absence of CsA. The slope of the line represents the *K*_i_. Data represent the mean ± S.E of three replicates.

The *PoxCYP18* gene was PCR amplified from the cDNA of *P. oxalicum* using gene-specific primers that resulted in an amplicon of 522 bp which was cloned into pET-28a(+) expression vector. The nucleotide sequence of the recombinant plasmid was confirmed by Sanger sequencing (accession no. MZ407579). The recombinant PoxCYP18, containing a six-histidine tag in its N-terminus, was heterologously expressed in *Escherichia coli* BL21(DE3)pLysS, followed by purification on Ni-NTA-agarose. SDS-PAGE analysis showed the presence of a single band of approximately 22 kDa, implying that the recombinant PoxCYP18 was purified to homogeneity (Fig. 1a). The purified PoxCYP18, after establishing its identity by immunoblotting with anti-His antibody (Fig. 1a), was used for estimation of PPIase activity by studying changes in the kinetics of chymotrypsin-mediated cleavage of the test peptide^29^. These studies revealed that compared to the uncatalyzed reaction (0.0140 ± 0.0033 s^−1^), the first-order rate constant was higher (0.077 ± 0.0017 s^−1^) in the presence of PoxCYP18 (Fig. 1b; Supplementary Fig. S3), and it increased with the amount of protein (Supplementary Fig. S4), signifying that this cyclophilin is an active PPIase. Bovine serum albumin (BSA), used as a negative control, had no significant effect on the reaction rate. The activity of FKBPs and cyclophilins is inhibited specifically by FK506 and CsA, respectively, with no cross-inhibition reported^8^. The presence of CsA resulted in a dramatic decrease in the rate constant of PoxCYP18 catalyzed reaction (0.0135 ± 0.0007 s^−1^; Fig. 1b; Supplementary Fig. S3, S5a), with an inhibition constant of 5.043 ± 1.13 nM (Fig. 1c), indicating abrogation of the PPIase activity. On the contrary, the addition of up to 2 µM FK506 had no significant effect on the enzymatic activity of PoxCYP18 (0.0735 ± 0.0014 s^−1^; Fig. 1b; Supplementary Fig. S3, S5a), further implying that this PPIase is a true cyclophilin.

### Role of cysteine residues in redox regulation of enzyme activity

**Figure 2:**
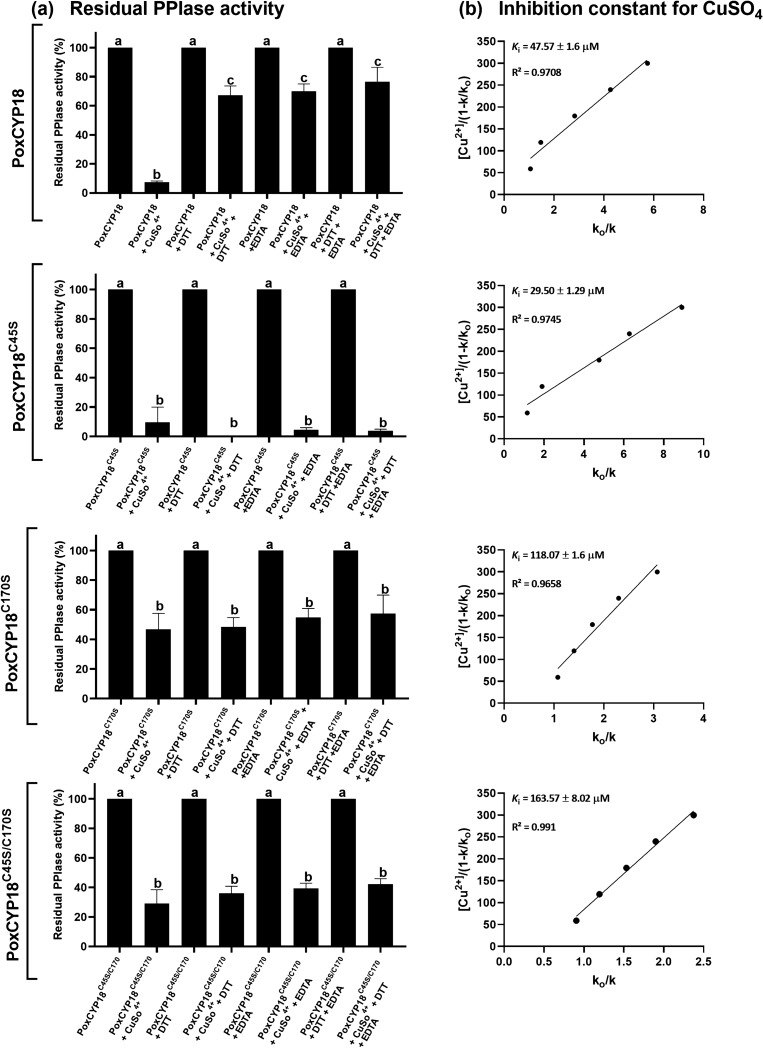
Analysis of redox regulation of the purified recombinant PoxCYP18 and its mutants PoxCYP18^C45S^, PoxCYP18^C170S^ and PoxCYP18^C45S/C170S^. (**a)** Effect of CuSO_4_ (300 µM), EDTA (1 mM) and DTT (10 mM) on peptidyl prolyl cis*–trans* isomerase (PPIase) activity of the native and the mutant PoxCYP18 proteins (4.4 µM each protein). The residual PPIase activity was calculated relative to the uninhibited controls. The values depict the mean of three replicates ± SE and the different lowercase letters denote significant difference between the treatments at P ≤ 0.001 (Tukey-HSD test; a = 0.05). (**b)** The Inhibition constant (*K*_i_) for Cu^2+^ was determined as gradient of the line of the best fit from a plot of [Cu^2+^]/(1 − *k*/*k*_0_) against *k*_0_/*k*, where *k* is the rate constant at any given Cu^2+^ concentration and *k*_0_ is the rate constant in the absence of Cu^2+^. The slope of the line represents the *K*_i_. The values depict the mean of three replicates ± standard error.

**Figure 3:**
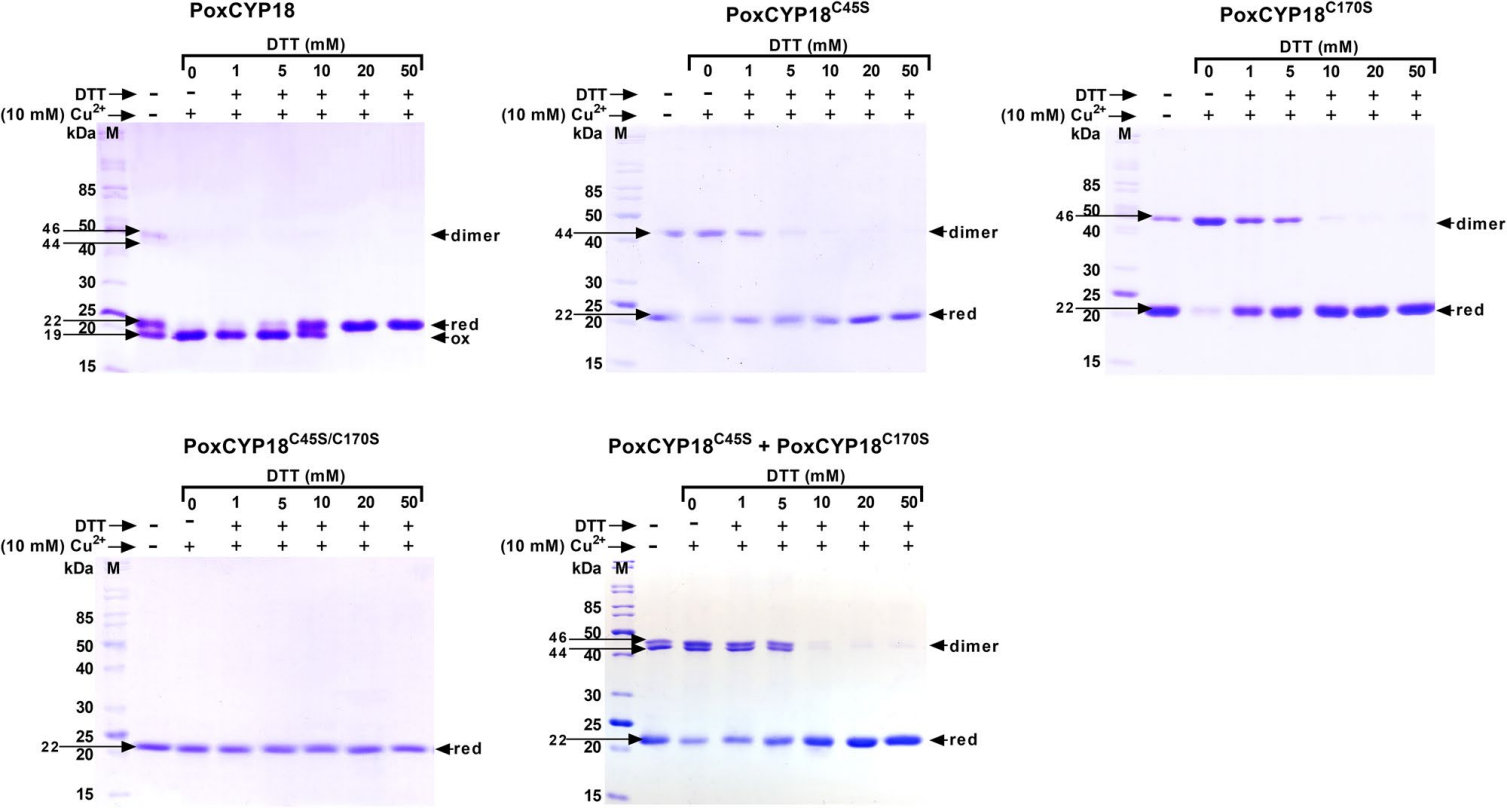
SDS-PAGE (12%) analysis of the purified PoxCYP18, PoxCYP18^C45S^, PoxCYP18^C170S^ and PoxCYP18^C45S/C170S^ proteins after titrating with reducing (0–50 mM DTT) and an oxidizing agent (10 mM Cu^2+^). The last panel depicts the electrophoretic pattern of mutant proteins PoxCYP18^C45S^ and PoxCYP18^C170S^ loaded in the same sample.

The PPIase activity of cyclophilins can be regulated in a redox-dependent or independent manner. Redox mechanisms involving disulfide bond formation between cysteine residues have been proposed to play an important role in controlling PPIase activity of different divergent and non-divergent cyclophilins^17,30^. The existence of a four or more amino acid long additional stretch in CLDs, which forms a protruding loop and corresponds to position 48–54 (XXGKXLH) in the wheat cyclophilin TaCYPA-1 (Supplementary Fig. S2), is a characteristic structural feature of the divergent cyclophilins distinguishing them from the non-divergent cyclophilins^31,32^. Divergent loop cyclophilins are generally observed in plants, such as TaCYPA-1 in wheat^33^, CsCYP in *Citrus sinensis*^34^ and Catr1 in *Catharanthus roseus*^35^. Two invariable cysteine residues (Cys-40 and Cys-168), and a conserved Glutamic acid (Glu-83) are also observed in TaCYPA-1, CsCYP and Catr1 that are unique to divergent cyclophilins^36^. On the contrary, the non-divergent cyclophilins, such as hCYPA, SmCYPA and Cpr1 from *Schistosoma mansoni* and *Saccharomyces cerevisiae*, respectively, lack the additional loop^30^. While the PPIase activity of a divergent cyclophilin, CsCYP, containing two cysteine residues at positions − 40 and − 168, is controlled by a loop displacement mechanism mediated through the formation of a disulfide bond between these residues^34^, the activity of a non-divergent cyclophilin, SmCYPA in *S. mansoni*, which contains four cysteine residues at positions − 69, − 122, − 126 and − 168, (Supplementary Fig. S2), is modulated through disulfide bond formation between Cys-122 and Cys-126^30^, with oxidation resulting in loss of activity in both the proteins. The PoxCYP18 is a non-divergent cyclophilin and consists of two highly conserved cysteine residues at Cys-45 and Cys-170 positions (Supplementary Fig. S2). CuSO_4_ has been employed as an oxidizing agent to study redox regulation in different cyclophilins^30,31,37^. In our study, the presence of 300 µM CuSO_4_ resulted in 95% abrogation of the PPIase activity of PoxCYP18, with an inhibition constant (*K*_i_) of 47.57 ± 1.6 µM (Fig. 2a, b). The PPIase activity of PoxCYP18 was not affected by either EDTA, a metal chelating agent, or DTT, that is used to reduce the disulfide bonds (Fig. 2a). To further understand the nature of Cu^2+^-induced inhibition, the Cu^2+^-treated PoxCYP18 was incubated with EDTA (1 mM) and DTT (10 mM) before the estimation of PPIase activity. The Cu^2+^-induced inhibition of PPIase activity of PoxCYP18 was partially reversed by EDTA and DTT since compared to 5% residual activity in the presence of Cu^2+^ alone, up to 70% and 75% residual activity was observed, respectively, when these two compounds were also added along with Cu^2+^ (Fig. 2a). Since PoxCYP18 consists of two cysteine residues at positions 45 and 170, we carried out a titration experiment of reducing versus oxidizing agent to investigate whether the loss of activity by Cu^2+^ was due to alteration in the structure. These studies revealed that migration of PoxCYP18 on 12% SDS-PAGE was redox-dependent (Fig. 3). Two closely moving bands of approximately 19 and 22 kDa were observed on 12% SDS-PAGE when PoxCYP18 was analyzed in the absence of both Cu^2+^ (oxidizing agent) and DTT (reducing agent) (Fig. 3). The 19 kDa and the 22 kDa bands are likely the oxidized and reduced forms of PoxCYP18 since the former was observed in the presence of Cu^2+^ alone (Fig. 3), and the latter at high concentrations of DTT (20 and 50 mM) (Fig. 3). These observations suggest that the recombinant PoxCYP18 produced in *E. coli,* under the conditions used, is a mixture of reduced and oxidized forms of this protein. SDS-PAGE analysis also revealed the presence of two additional bands of 44 and 46 kDa, respectively, for the unmutated PoxCYP18. Immunoblotting with anti-His antibodies confirmed the identity of all the bands as either monomers of PoxCYP18 (19 kDa and 22 kDa) or its dimers (44 and 46 kDa) (Supplementary Fig. S6). However further analysis is required to confirm the dimeric nature of PoxCYP18 by employing gel filtration and other biophysical techniques.

### Generation of PoxCYP18 variants

To investigate the functional significance of cysteine residues in redox regulation of PoxCYP18, we generated site-directed mutants of PoxCYP18 by substituting cysteine residues with serine at positions − 45 (PoxCYP18^C45S^), − 170 (PoxCYP18^C170S^), and at both − 45 and − 170 (PoxCYP18^C40S/C170S^). The recombinant mutant proteins were purified and validated by SDS-PAGE analysis (Fig. 1a), followed by western blotting with anti-His antibody (Supplementary Fig. S6). Enzymatic analysis revealed that relative to the uncatalyzed control, the first-order rate constant was significantly higher in the presence of PoxCYP18^C45S^ (0.0657 ± 0.0028 s^−1^), PoxCYP18^C170S^ (0.0741 ± 0.004 s^−1^) and PoxCYP18^C40S/C170S^ (0.0350 ± 0.0016 s^−1^), respectively (Fig. 1b; Supplementary Fig. S3), while BSA had no significant effect. The rate of reaction increased with an increase in the amount of the respective mutant proteins (Supplementary Fig. S4). These observations indicate that the observed PPIase activity was specifically due to the presence of mutant cyclophilins. The *k*_cat_/*K*_m_ values for PoxCYP18^C45S^ (1.18 × 10^7^ M^−1^ s^−1^) and PoxCYP18^C170S^ (1.40 × 10^7^ M^−1^ s^−1^) were similar to the native PoxCYP18 (1.46 × 10^7^ M^−1^ s^−1^). However, *k*_cat_/*K*_m_ of the double mutant PoxCYP18^C40S/C170S^ was significantly lower (6.4 × 10^6^ M^−1^ s^−1^), implying that though either of the two cysteines are dispensable for *cis–trans* isomerase activity of this protein, the simultaneous substitution of these residues affect the enzymatic activity adversely. The presence of CsA resulted in a dramatic decrease in the rate constant for all three mutant proteins (Supplementary Fig. S5b–d) with *K*_i_ values of 6.47 ± 0.06 nM, 7.85 ± 1.23 nM and 6.08 ± 0.08 nM for PoxCYP18^C45S^, PoxCYP18^C170S^, and PoxCYP18^C40S/C170S^, respectively (Fig. 1c) being comparable to PoxCYP18 (5.04 ± 1.13 nM).

To investigate how the substitution of cysteine residues in PoxCYP18 affects redox regulation, the PPIase activity of these mutant proteins was also studied after treatment with Cu^2+^. It was observed that the substitution of Cys170 alone or simultaneously with Cys45 resulted in a decrease in sensitivity to Cu^2+^. This was evident since *K*_i_ values of Cu^2+^ were significantly higher for PoxCYP18^C170S^ (118.07 ± 1.6 µM) and PoxCYP18^C40S/C170S^ (163.57 ± 8.02 µM) compared to the unmutated PoxCYP18 (*K*_i _= 47.57 ± 1.6 µM; Fig. 2b). Contrary to the native PoxCYP18, the addition of EDTA and DTT did not revert the Cu^2+^-induced inhibition of any of the PoxCYP18 mutants (Fig. 2a).

The effect of mutating the cysteine residues on structural changes was studied by electrophoretic analysis. It was observed that compared to native PoxCYP18, the mutant proteins depicted an altered migration pattern on SDS-PAGE (Fig. 3). Only a single band, that corresponded to a reduced band of native PoxCYP18 (22 kDa), was observed for all the mutant PoxCYP18 proteins under both oxidizing and reducing conditions (Fig. 3). The PoxCYP18^C45S^ and PoxCYP18^C170S^ depicted bands of 44 and 46 kDa, respectively, which correspond to the two bands observed for native PoxCYP18 (Fig. 3). Immunoblotting confirmed the high molecular weight bands of 44 and 46 kDa as dimers of these mutants (Supplementary Fig. S6). These bands, however, disappeared under reducing conditions implying the role of cysteine residues in the formation of dimers (Fig. 3). The bands of 44 and 46 kDa appear to be the dimers due to inter-monomer disulfide bond between − 170 cysteine residues of PoxCYP18^C45S^, and − 45 cysteine residues of PoxCYP18^C170S^, respectively, since loading of both these mutants together depicted the presence of two bands compared with one each when these proteins were analyzed separately by SDS-PAGE (Fig. 3). The fact that the double mutant PoxCYP18^C45S/C170S^ did not depict a dimer band under any of the conditions (Fig. 3) further supports this inference.

### Effect of NaCl and temperature on enzyme activity

**Figure 4:**
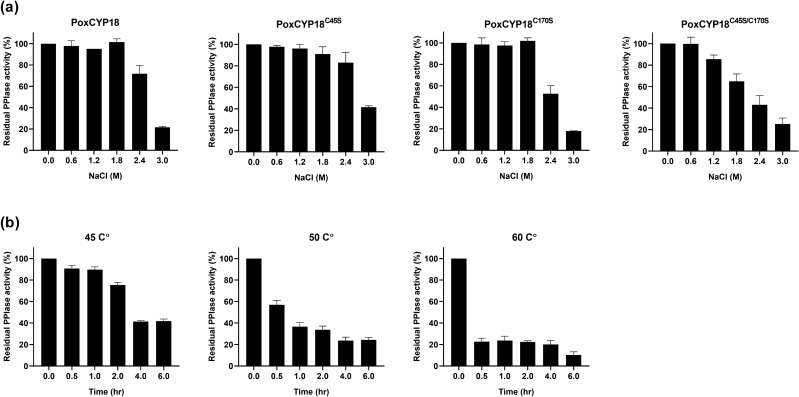
**(a)** Effect of salt (NaCl) on peptidyl-prolyl *cis–trans* isomerase (PPIase) activity of PoxCYP18 and its mutants PoxCYP18^C45S^, PoxCYP18^C170S^ and PoxCYP18^C45S/C170S^. (**b)** Effect of temperature on PPIase activity of PoxCYP18 at various temperatures. The mutant proteins PoxCYP18^C45S^, PoxCYP18^C170S^ and PoxCYP18^C45S/C170S^ lost their PPIase activity completely after 30 min at 45 °C due to which this data has not been included in the figure. Data represent the mean ± S.E of three replicates.

As the strain of *P. oxalicum*, the source of PoxCYP18, is a halotolerant fungus and can tolerate up to 3 M NaCl, we therefore also analyzed the stability of this protein under high salt and temperature (45 °C, 50 °C and 60 °C) conditions. In vitro biochemical assays revealed that PPIase activity of PoxCYP18, PoxCYP18^C45S^ and PoxCYP18^C170S^ was stable in up to 1.8 M NaCl, with the significant decrease observed only at 2.4 M (72% residual activity) and 3.0 M (22% residual activity: Fig. 4a). However, the double mutant PoxCYP18^C45S/C170S^, showed greater sensitivity to salt since the decrease in activity was observed from 1.2 M NaCl onwards (Fig. 4a). Thermostability analysis demonstrated the presence of approx. 70% residual PPIase activity after 2 h at 45 °C, compared to 57% and 25% after 30 min at 50 °C and 60 °C, respectively. Retention of approximately 38%, 22% and 11% residual activity even after 6 h at 45 °C, 50 °C and 60 °C, respectively, suggests that PoxCYP18 is relatively thermostable (Fig. 4b). On the contrary, all the mutants (PoxCYP18^C45S^, PoxCYP18^C170S^ and PoxCYP18^C45S/C170S^) of this protein lost their PPIase activity by 30 min at 45 °C, indicating the role of cysteine residues in the stability of this cyclophilin.

### Stress tolerance in *E. coli*

**Figure 5:**
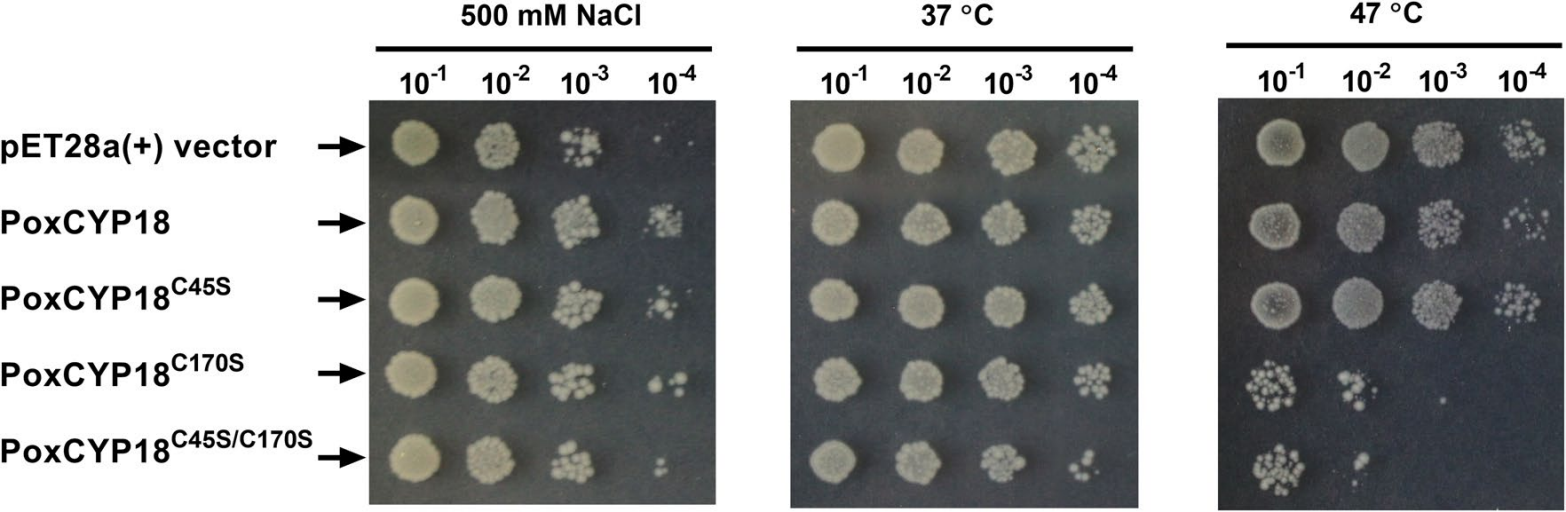
Effect of PoxCYP18 and its mutants, PoxCYP18^C45S^, PoxCYP18^C170S^ and PoxCYP18^C45S/C170S^, on salt (500 mM) and heat (47 °C) stress tolerance of *E. coli* BL21(DE3)pLysS cells. The growth of the *E. coli* BL21(DE3)pLysS cells transformed with *PoxCYP18* and its mutants was compared on LB media relative to controls transformed with non-recombinant pET-28a(+).

The cyclophilins have been implicated in abiotic stress tolerance of bacteria, fungi and plants^27,28,38,39^ but the role of PoxCYP18 has not been studied in stress adaptation. Therefore, in the present study, we investigated the role of PoxCYP18 in heat and salt stress using *E. coli* as a model. The protective role of PoxCYP18 in *E. coli* was explored in response to salt stress (500 mM NaCl) by inducing the protein with IPTG and following the growth of cultures at 37 °C by taking absorbance at 600 nm (A_600_) until stationary phase (Supplementary Fig. S7), and also by spot analysis (Fig. 5; Supplementary Fig. S8). It was observed that compared to control (*E. coli* cells transformed with empty vector), the PoxCYP18 expressing cells showed a higher number of colonies, signifying enhanced tolerance to salt stress (Fig. 5; Supplementary Fig. S8). Though the cells expressing mutant proteins PoxCYP18^C45S^, PoxCYP18^C170S^ and PoxCYP18^C45S/C170S^ exhibited lesser growth compared to PoxCYP18, it was still higher than the control cells containing the non-recombinant vector. These results indicate that the mutants also imparted tolerance under salt stress, albeit to a lesser extent than the native PoxCYP18 (Fig. 5). The protective role of PoxCYP18 in *E. coli* was also studied against heat stress (47 °C). Relative to control, no significant difference was observed at high temperatures in the growth of *E. coli* cells expressing PoxCYP18 and PoxCYP18^C45S^. However, the growth was adversely affected at both 37 °C and 47 °C when the *E. coli* cells were transformed with PoxCYP18^C170S^ and PoxCYP18^C45S/C170S^, implying that these mutants have deleterious effects (Fig. 5).

### 3D structure modeling and molecular dynamics (MD) simulation

**Figure 6:**
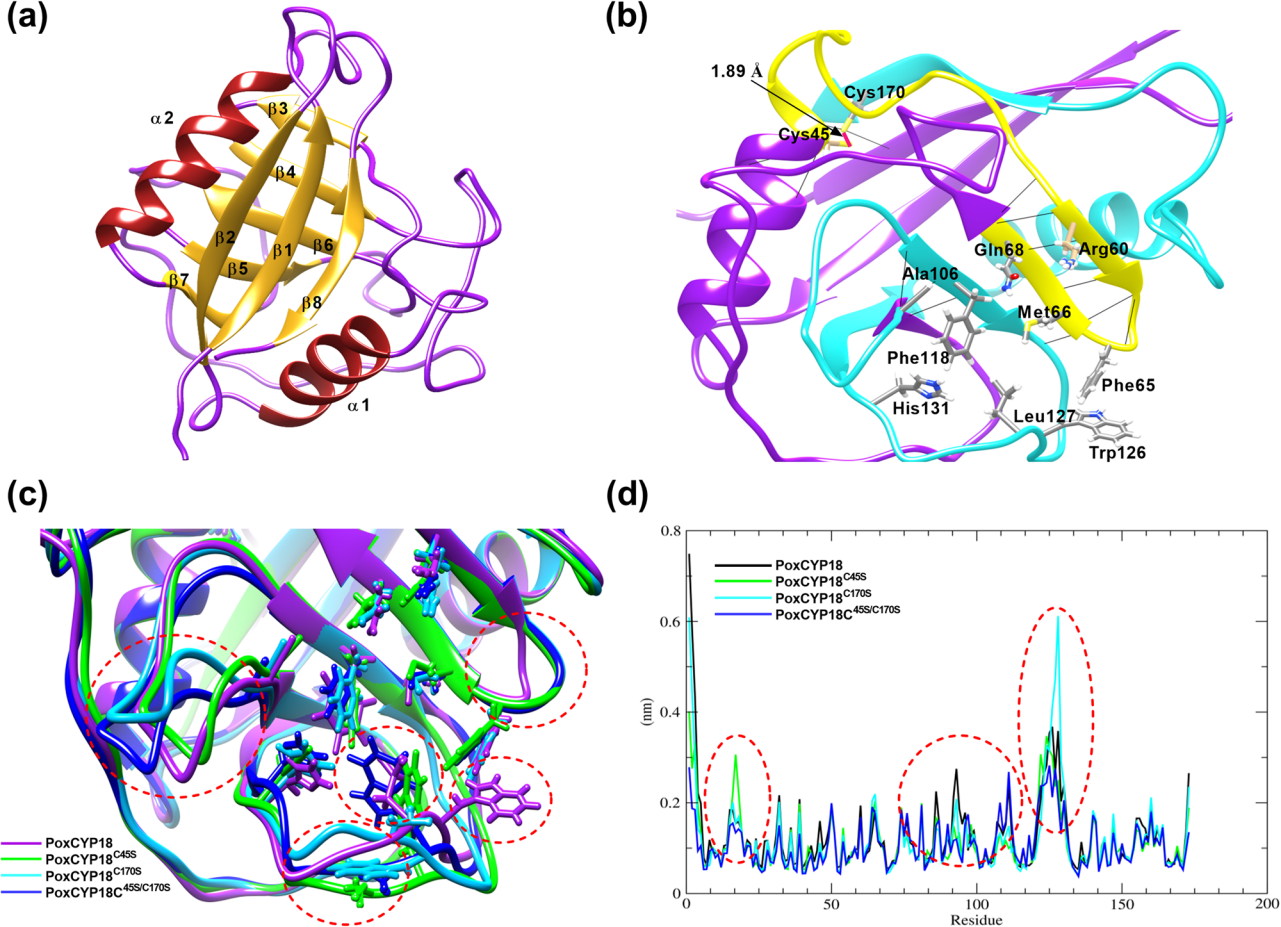
Analysis of 3D structure of PoxCYP18 and its different mutants. (**a)** An overview of 3D structure of PoxCYP18 showing secondary structure elements. (**b)** PoxCYP18 with a focused view of active site residues and secondary structure segments harboring different residues. Yellow colored segment depicts the presence of Cys45 and the active site residues Arg60, Phe65, Met66 and Gln68. Cyan colored segment contains Cys170 and the active site residues viz., Ala106, Phe118, Trp126, Leu127 and His131. (**c)** 3D structure alignment of PoxCYP18 (purple), PoxCYP18^C45S (^green), PoxCYP18^C170S^ (cyan) and PoxCYP18^C45S/C170S^ (blue). The overall 3D structure is conserved in all the models, with differences observed in the loop regions, specifically, the one containing Phe118, Trp126, Leu127 and His131 amino acids (circled red). (**d)** Comparison of root mean fluctuation plots of amino acid residues in PoxCYP18 (black), PoxCYP18^C45S^ (green), PoxCYP18^C170S^ (cyan) and PoxCYP18^C45S/C170S^ (blue).

The PPIase activity of cyclophilins is governed in a redox-dependent as well as independent manner. The redox-dependent mechanisms are different for the divergent and non-divergent cyclophilins, and involve precise control of PPIase activity through the formation of disulfide bonds between cysteine residues^17,30^. The results of the present study revealed that the activity of PoxCYP18 is regulated through redox mechanisms, prompting us to generate 3D models of PoxCYP18 and its mutants (PoxCYP18^C45S^, PoxCYP18^C170S^ and PoxCYP18^C45S/C170S^), followed by 50 ns MD simulations for gaining better insight into the possible structural features that may be responsible for this modulation. 3D modeled structure of PoxCYP18 (Fig. 6a) shows the presence of a CLD (16–172 residues) consisting of eight β sheets and two α helixes. All the nine active site residues responsible for CsA binding and PPIase activity are conserved (Fig. 6b), and the two cysteine resides that are well placed (1.89 Å**)** and oriented towards each other (Fig. 6b), indicate a possibility of forming disulfide bond under oxidizing conditions. Importance of these two cysteine residues in maintaining the overall stability of the PoxCYP18 structure and their effect on the dynamics of the active site can be understood from their presence in two important segments. The first segment (yellow color, Fig. 6b) contains Cys-45 and the active site residues Arg60, phe65, Met66 and Gln68, while the second segment (cyan color; Fig. 6b) contains Cys-170 and the active site residues Ala106, Phe118, Trp126, Leu127 and His131. Most of the active site residues (Arg60, Met66 and Gln68) in the first segment are part of the beta-sheet stabilized by a strong network of H-bonds. On the other hand, the majority of the active site residues (Ala106, Trp126, Leu127 and His131) in the second segment are part of the loop region making them more prone to fluctuations, thereby, rendering more dynamics to the active site. The presence of cysteine residues at the ends of these two segments might be significant for maintaining the overall active conformation of the protein and also for controlling the active site dynamics, specifically involving the residues Trp126, Leu127 and His131. His131is essential for PPIase activity and changes in this residue have been reported to completely abrogate the catalytic activity of this enzyme^40^.

Although PoxCYP18 is a non-divergent cyclophilin, the arrangement of Cys-45 and Cys-170 residues is similar to that observed for divergent cyclophilin, CsCYP, containing two cysteine residues at positions − 40 and − 168^34^ (Supplementary Fig. S2). To study the role of these residues in PoxCYP18, three mutant structures PoxCYP18^C45S^, PoxCYP18^C170S^ and PoxCYP18^C45S/C170S^ were also modeled and subjected to 50 ns MD simulations (along with the native PoxCYP18) using Gromacs. The average structures of all the PoxCYP18 variants obtained though the MD simulations were superimposed (Fig. 6c), which indicated that most of the secondary structure elements are conserved between the native and the mutant forms. However, some of the loop regions were observed to vary significantly, including the two loop regions belonging to the segments harboring the active site residues (for example Ala106, Trp126, Leu127 and His131, Fig. 6c, highlighted in red circles) and cysteine residues at their ends. Relative to PoxCYP18, fluctuations in these loop regions of all three mutants were further confirmed by the root mean square fluctuation values of the amino acids during the time of simulation. The superimposition of the active site residues (Fig. 6c) suggested that the average orientation of PoxCYP18^C45S^ active site residues, specifically the Trp126 (green color), makes the active site less accessible for substrate binding. The Trp126 residue (blue color) in the double mutant, PoxCYP18^C45S/C170S^, seems to orient further inwards towards the active site, rendering it even less accessible to the substrate. Such conformations of the active site residues in PoxCYP18^C45S^ and PoxCYP18^C45S/C170S^ might explain the decrease in catalytic efficiencies (1.18 × 10^7^ M^−1^ s^−1^ and 6.4 × 10^6^ M^−1^ s^−1^, respectively) relative to the PoxCYP18 (1.46 × 10^7^ M^−1^ s^−1^). On the contrary, in spite of some of the residues showing greater fluctuations in the loop regions (residue no. 115–135, Fig. 6d), PoxCYP18^C170S^ is able to keep the active site residues (cyan) well-oriented, which may enable the substrate to access the active site (Fig. 6c), thus leading to comparable catalytic efficiency (1.40 × 10^7^ M^−1^ s^−1^) with the native PoxCYP18 (1.46 × 10^7^ M^−1^ s^−1)^. These analyses imply that the cysteine resides not only makes the PoxCYP18 protein responsive to redox conditions, but also plays an important role in maintaining the overall structure of the active site open for access to the substrate.

The changes in the overall compactness of the 3D structures were also analyzed by measuring the radius of gyration (Supplementary Fig. S9). According to this analysis, the overall structure of the mutants PoxCYP18^C45S^, PoxCYP18^C170S^ and PoxCYP18^C45S/C170S^ showed some fluctuations in their 3D structures during the time of simulation. As compared to PoxCYP18, which maintains the overall native structure, all three mutants tend to deviate more frequently from their overall starting shape. In the native PoxCYP18, the formation of a disulfide bond between the two cysteine residues under oxidized conditions may make the structure more compact and stable as compared to the reduced state, explaining the altered migration pattern of the mutant proteins on SDS-PAGE. This hypothesis, however, requires validation at the structural level by further biophysical characterization of these proteins.

## Discussion

Cyclophilins are conserved across prokaryotes and eukaryotes, and have been implicated in several cellular functions, such as protein trafficking and folding, receptor signaling, RNA processing, immunological response, spliceosome assembly, and plant growth and development, and stress adaptation^28,41–45^. Cyclophilins have also been demonstrated to play important roles in different fungal species. Some of the functions ascribed to cyclophilins in fungi include maintenance of growth and virulence in pathogenic yeast, *C. neoformans*^23^, protein folding and tolerance to heat stress in *S. cerevisiae*^46–53^. Besides, heterologous expression of cyclophilin genes from *Piriformospora indica* (*PiCYPA)* and *Porocentrum lilacinum (PlCYP4* and *PlCYP6)* was also shown to confer tolerance to different abiotic stresses in transgenic plants and *E. coli*^54,55^*.* Furthermore, stress-induced upregulation of cyclophilin genes has also been demonstrated in several other microbes, implying their role in adaptation to adverse environmental conditions^56–60^.

Previous studies from our laboratory revealed that PPIase activity in the mycelia of a halotolerant strain of *P. oxalicum* was enhanced significantly in the presence of salt. These studies further showed that cyclophilins contributed to the stress-induced increase in mycelial PPIase activity, with one of the genes, *PoxCYP18*, showing significant upregulation^28^. Until this observation, the characterization of PPIase activity had not been reported in any of the *Penicillium* species. In the present study, we carried out cloning of cDNA (522 bp) of PoxCYP18, followed by its biochemical characterization. PoxCYP18 consists of 173 AAs, and is a cytosolic protein of 18.9 kDa that shows 70% similarity at amino acid level with human cyclophilin, hCYPA. PoxCYP18 comprises a single CLD, with all the amino acid residues critical for PPIase activity and CsA binding being conserved. PoxCYP18 demonstrated PPIase activity which was inhibited only by the specific inhibitor, CsA, while Fk506 had no apparent effect (Fig. 1b; Supplementary Fig. S3, S5), implying that PoxCYP18 is a true cyclophilin. The catalytic efficiency (*k*_cat_/*K*_m_) of PoxCYP18 (1.46 × 10^7^ M^−1^ s^−1^) is comparable with values reported for cyclophilins from other species such as *S. cerevisiae* (Cpr1; 1.52 × 10^7^ M^−1^ s^−1^), human (hCYPA; 1.4 × 10^7^ M^−1^ s^−1^) and *Zea mays* (PPI; 1.1 × 10^7^ M^−1^ s^−1^)^61–63^. The inhibition constant of CsA for PoxCYP18 (5.04 nM) is consistent with the values observed for other cyclophilins from *Leishmania major* (5.2 nM)^64^, *Toxoplasma gondii* (5.0 nM)^65^, *Vicia faba* (3.9 nM)^66^ and *Z. mays* (6.0 nM)^63^, but lower than that reported for Cpr1(40 nM) and Cpr2 (101 nM) from *S. cerevisiae*^61^, TaCYPA-1 from *T. aestivum* (78.3 nM)^33^ and human cyclophilin hCYPD (300 nM)^67,68^, thus, signifying the variability in sensitivity of different cyclophilins to CsA.

PPIase activity of several cyclophilins, such as SmCYPA, CsCYP and TaCYPA-1, which are regulated through redox mechanisms, is inhibited by oxidizing agents such as CuSO_4_^30,31,34,69^. Redox mechanisms involving disulfide bond formation between cysteine residues, that modulate PPIase activity, are different for divergent and non-divergent cyclophilins^17,30^. Multiple sequence alignment of PoxCYP18 with the previously reported divergent and non-divergent cyclophilins showed that PoxCYP18 is a non-divergent cyclophilin and consists of two highly conserved cysteine residues at positions 45 and 170 (Supplementary Fig. S2). Treatment with CuSO_4_ resulted in inhibition of the PPIase activity of PoxCYP18, with an inhibition constant of 47.57 ± 1.6 µM. The CuSO_4_-induced inhibition of PoxCYP18 was attenuated by the chelating agent EDTA and reducing agent DTT, thus supporting the role of redox mechanisms in the regulation of this cyclophilin. To further understand the importance of these two cysteine residues in the regulation of PPIase activity of PoxCYP18, site-directed mutants were generated by substituting the cysteine at these positions, alone or together, with serine residue. Substitution of either Cys-45 and Cys-170 alone did not affect the catalytic efficiency, since *k*_cat_/*K*_m_ values for the PoxCYP18^C45S^ (1.18 × 10^7^ M^−1^ s^−1^) and PoxCYP18^C170S^ (1.40 × 10^7^ M^−1^ s^−1^) were similar to the native PoxCYP18 (1.46 × 10^7^ M^−1^ s^−1^). However, the substitution of both the cysteine residues simultaneously resulted in a significant decrease in catalytic efficiency of PoxCYP18^C45S/C170S^ (6.4 × 10^6^ M^−1^ s^−1^) which might be due to disruption of enzymatic structure, as also supported by the computational analysis (Fig. 6c). Furthermore, our studies also showed that the cysteine residues are not implicated in CsA inhibition as *K*_i_ values were similar for the native PoxCYP18 and its three mutants (Fig. 1c). Of the two cysteine residues Cys-45 and Cys-170, the latter appears to play a major role in Cu^2+^ sensitivity, since the substitution of this residue with serine in single (PoxCYP18^C170S^) and double mutant (PoxCYP18^C45S/C170S^) resulted in substantial increase in *K*_i_ (118.07 ± 1.6 µM and 163.57 ± 8.02 µM, respectively), indicating a decrease in sensitivity as compared to the native PoxCYP18 (*K*_i_ = 47.57 ± 1.6 µM) and PoxCYP18^C45S^ (*K*_i_ = 29.50 ± 1.29 µM). Earlier studies had shown that the cysteine residues at 40 and 168 position in *Caenorhabditis elegans* cyclophilin, CYP3; Cys-52 and Cys-181 in hCYP40, and Cys-122 and Cys-126 in TaCYPA-1 could form a disulfide bond under oxidizing conditions^69–71^. The Cu^2+^ -induced loss of activity in CYP3 was attributed to an alteration in the structure due to the formation of a disulfide bridge between Cys-40 and Cys-168^34^. Therefore, to understand the role of structural changes in Cu^2+^-induced inhibition, the PoxCYP18 was subjected to SDS-PAGE analysis under different oxidative and reducing conditions. SDS-PAGE analysis in the absence of both DTT and CuSO_4_ depicted the presence of two closely moving bands of 19 and 22 kDa, and of 44 and 46 kDa. The 19 kDa and 22 kDa bands correspond to fully oxidized and reduced forms of PoxCYP18, respectively, since these were the only bands observed under completely oxidative (10 mM CuSO_4_) and reductive conditions (50 mM DTT), signifying confirmational changes that resulted in altered migration. On the contrary, the mobility of either of the three mutants was unaffected by the oxidizing conditions and only a single band corresponding to the reduced state (22 kDa) was observed. These observations indicate the role of disulfide linkages in maintaining the structural confirmation of PoxCYP18. Immunoblotting studies suggested that the bands corresponding to 44 and 46 kDa proteins are likely the dimers of PoxCYP18 formed through intermolecular disulfide linkages between two polypeptides under an oxidizing environment in *E. coli*. Further, the 44 kDa band is the result of dimerization through Cys-170 of the two polypeptides while 46 kDa band is formed due to disulfide bond linkages between Cys-45. As the source of PoxCYP18, *P. oxalicum*, is a halotolerant fungus that can tolerate up to 3 M NaCl, we also investigated the ability of this protein to impart tolerance in *E. coli*. Compared to the control, substantially higher growth of *E. coli* cells expressing PoxCYP18 in the presence of 500 mM NaCl implied that this cyclophilin is capable of conferring protection to a biological system against salt stress. However, the expression of this cyclophilin did not provide tolerance under higher temperature (47 °C), suggesting this protein's stress-specific role. Several cyclophilins, such as hCYPD, PfCYP19A, and CPR6 and CPR7 from humans, *Plasmodium falciparum* and *S. cerevisiae*, respectively, have been reported to possess chaperonic activity in addition to PPIase activity^67,68,72,73^. It is likely that PoxCYP18 might be imparting salt tolerance to *E. coli* by facilitating the folding of cellular proteins through these activities which are stable in the presence of high salt of up to 1.8 M (Fig. 4a). However, further studies that include the analysis of chaperonic activities and identification of target proteins are required to elucidate the molecular mechanism of PoxCYP18-induced stress tolerance.

## Materials and methods

### Bioinformatics analysis

The amino acid sequences of cyclophilins from different species were retrieved from the NCBI server (http://www.ncbi.nlm.nih.gov/) using their reported accession numbers. Pairwise percentage sequence identity and similarity were calculated using the Matrix Global Alignment Tool (MatGAT) version 2.02 using a BLOSUM50 scoring matrix. Multiple sequence alignment of amino acid sequences of different cyclophilins was performed using the MUSCLE algorithm in Jalview software 2.11.1.3. with default parameters (http://www.jalview.org/). Secondary structure features were predicted using the Jpred3 server (http://www.compbio.dundee.ac.uk/www-jpred/). The phylogenetic tree was constructed with the aligned cyclophilin sequences using the ClustalW algorithm in Mega X software and the neighbor-joining (NJ) method with default options. For statistical reliability, the bootstrap analysis was conducted with 1000 replicates.

### Cloning of cDNA encoding cyclophilin PoxCYP18 in *Penicillium oxalicum*

Previous studies in our lab showed that a gene encoding an 18.9 kDa cyclophilin PoxCYP18 in *P. oxalicum* depicted significantly gene higher expression under salt stress (15% NaCl)^28^. In the present study, the cDNA encoding this protein was cloned and heterologous expression and protein purification was carried out for its further characterization. Total RNA was extracted from four days old mycelia tissues of *P. oxalicum* using TRIzol (Invitrogen, USA) according to the manufacturer's instructions. After removing DNA by DNaseI (Sigma-Aldrich) treatment, the RNA was quantified, and its integrity was confirmed by denaturing agarose gel electrophoresis (1.4%) followed by staining with ethidium bromide. Superscript III First-strand synthesis system kit (Invitrogen) was used to synthesize cDNA from 5 μg of total RNA with random hexamer primers. The full-length amplification of the *PoxCYP18* gene by PCR was carried out using the cDNA of *P. oxalicum* as a template (100 ng) in a 20 μl reaction volume that contained 0.4 μM gene-specific forward and reverse primers (Supplementary Table 1), Q5 DNA polymerase (0.02 U/µl), 1X Q5 buffer, and 0.2 mM of each dNTP under the following conditions: 98 °C for 2 min, followed by 35 cycles of 98 °C for 10 s, 58 °C for 30 s, 68 °C for 30 min, and a final extension at 68 °C for 5 min. The purified PCR product was cloned into the pET-28a(+) vector (Novagen) using *Bam*HI and *Eco*RI restriction sites. The pET-28a-PoxCYP18 recombinant plasmid sequencing using the T7 promoter region primers was outsourced to Bioserve Biotechnologies (I) Pvt. Ltd., India. After sequence confirmation the nucleotide sequence of PoxCYP18 gene was deposited in the GenBank data base with accession number of MZ407579.1.

### Site-directed mutagenesis of *PoxCYP18*

Site-directed mutants of *PoxCYP18* were generated by replacing cysteine (C) residues with serine (S) at positions 45 (*PoxCYP18*^*C45S*^), 170 (*PoxCYP18*^*C170S*^), and 45/170 (*PoxCYP18*^*C45S/C170S*^), using the site-specific PCR primers (Supplementary Table 1). The recombinant pET-28a( +) containing *PoxCYP18* gene was used as a template for PCR amplification. The PCR mixture contained 1 µM forward and reverse primers, 200 μM dNTPs, 1 × Q5 buffer, 60 ng template DNA and Q5 DNA polymerase (0.02 U µl^−1^). PCR amplification was carried out with initial denaturation at 98 °C for 2 min, followed by 35 cycles of denaturation at 98 °C for 10 s, annealing at 54 °C (for C45S and C45S/C170S double substitution) and 58 °C (for C170S substitution), and extension at 72 °C for 3 min for 35 cycles, with a final extension at 72 °C for 5 min. The amplified products were treated with the restriction enzyme *Dpn*I (Fermentas, USA) to remove the parental plasmid DNA. The 20 µl reaction mixture, containing 9 µl amplified product, 1X Tango buffer and 0.2 µl *Dpn*I (2U), was incubated at 37 °C for 30 min. After *Dpn*I digestion, 2 µl of the reaction mix was used for the transformation of the competent *E. coli* DH5α cells. The point mutations were confirmed by sequencing the recombinant plasmids.

### Induction and purification of the recombinant proteins

The native PoxCYP18 and its mutants were expressed as 6 × His fusion proteins in *E. coli* BL21(DE3)pLysS cells after induction of cultures with 1 mM isopropyl-β-d-1-thiogalactopyranoside (IPTG) when A_600_ reached 0.4–0.5, followed by further incubation at 25 °C for 4 h with shaking at 200 rpm. The induced proteins were analyzed by 12% SDS-PAGE followed by staining with Coomassie Brilliant Blue-R250 (CBB)^74^. For purification of the recombinant proteins, the cells were lysed in lysis buffer [50 mM Tris-HCl (pH 8.0), 300 mM NaCl, 0.25% Triton X-100, 1 mM protease inhibitor cocktail (PIC), 10% glycerol, 10 mM imidazole] followed by sonication (time: 2 min, pulse on: 9.0 s/off: 5.0 s). The lysed samples were centrifuged for 20 min at 13,000 rpm, and the supernatants containing the recombinant fusion proteins were incubated with Ni-NTA slurry (G Biosciences, USA) in binding buffer [50 mM Tris-HCl (pH 8.0), 300 mM NaCl, 0.25% Triton X-100, 1 mM PIC, 10% glycerol, 10 mM imidazole] for 1 h at 4 °C and applied on to the columns. The columns were washed with three bed-volumes of wash buffer [50 mM Tris–HCl (pH 8.0), 300 mM NaCl, 0.25% Triton X-100, 1 mM PIC, 10% glycerol, 50 mM imidazole]. The matrix-bound proteins were eluted thrice by addition of one bed-volume of elution buffer [50 mM Tris-HCl (pH 8.0), 300 mM NaCl, 0.25% Triton X-100, 1 mM PIC, 10% glycerol, 250 mM imidazole]. The purified recombinant proteins were separated by 12% SDS-PAGE under reducing conditions.

### Immunoblot analysis of recombinant proteins

Immunoblotting of the purified recombinant proteins was carried out as described by Sambrook et al. (1989)^75^. The proteins were separated by 12% SDS-PAGE and transferred to Hybond C membrane (Amersham Pharmacia Biotech, England) using transfer buffer [150 mM glycine, 20 mM Tris-HCl (pH 8.0), 0.1% SDS, 10% methanol]. The membrane, after staining with Ponceau S (Sigma-Aldrich, USA) and destaining in autoclaved double distilled water, was incubated for 2 h at room temperature in blocking buffer [200 mM Tris-HCl (pH 7.5), 150 mM NaCl, 0.02% Tween-20, 5% skimmed milk). The blots were incubated with mouse anti-His antibodies (Sigma-Aldrich, USA) at 1:1,000 dilution in blocking buffer for overnight. Following washing thrice with TBST buffer [200 mM Tris-HCl (pH 7.5), 150 mM NaCl, 0.02% Tween-20] for 10 min each, the blots were incubated in horse radish peroxidase-conjugated goat anti-mouse secondary antibodies (Sigma-Aldrich, USA; diluted 1:80,000 in TBS buffer) for 2 h. After washing the blot thrice with TBST for 10 min each, the protein-antibody complex was visualized by incubating it in a 3,3′-diaminobenzidine solution.

### Reducing and non-reducing SDS-PAGE to visualize disulfide bond formation

20 μg of the native PoxCYP18 and the mutated proteins were pretreated with an oxidizing agent (CuSO_4_, 10 mM) for 15 min at room temperature. The CuSO_4_-treated proteins were divided into 20 μg aliquots to which varying amounts of dithiothreitol (DTT; 1–50 mM) was added. After adding SDS sample buffer [60 mM Tris-HCl (pH 6.8), 2% SDS, 10% glycerol, 0.02% bromophenol blue] lacking reducing agent, the aliquot was directly loaded onto a 12% SDS-PAGE and stained with CBB^74^.

### Estimation of peptidyl-prolyl *cis–trans* isomerase (PPIase) activity

PPIase activity of the different PoxCYP18 proteins was determined using a chymotrypsin-based coupled reaction at 15 °C for 360 s^29^. Chymotrypsin has high substrate specificity for the *trans*-isomer of the test peptide containing proline (*N*-succinyl-ala-ala-pro-phe-*p*-nitroanilidine) but not the *cis*-isomer. Approximately 88% of the *N*-succinyl-ala-ala-pro-phe-*p*-nitroanilide (Sigma; S-7388), consisted of *trans*-Ala-Pro bond at equilibrium in solution, with the remaining in the *cis* form. The 88% of the peptide substrate present in the *trans* form is cleaved spontaneously in mixing time. The rate constant is calculated for the remaining 12% *cis* form, which is cleaved upon enzymatic conversion to the *trans* form by PPIase^76^. The 1 ml assay mixture contained 80 µM succinyl-ala-ala-pro-phe-*p*-nitroanilidine as test peptide, assay buffer [50 mM HEPES (pH 8.0), 150 mM NaCl, 0.05% Triton X-100] and different concentrations of the purified proteins. The reaction was initiated by the addition of chymotrypsin at a final concentration of 300 µg/ml. The absorbance change at 390 nm was monitored at 15 °C with a Spectrophotometer (Perkin-Elmer Lambda Bio 25) equipped with a Peltier temperature control system. The data obtained were analyzed using Grafit 4.0 software (http://www.erithacus.com/grafit). The rate constant was calculated by GraFIT4 software using the first-order offset equation “y = Limit × (1 − exp^(−k×t)^) + Off” (with *k* as the observed rate constant *k*_obs_). The PPIase activity was calculated as the product of the difference in the catalyzed and uncatalyzed first-order rate constants (derived from the kinetics of the absorbance change at 390 nm) and the amount of substrate in each reaction^77^. The rate constant of the isomerase was determined by subtracting *k*_0_ from *k*_obs_ (with *k*_0_ as the spontaneous uncatalyzed *cis–trans* isomerization rate) and these values were plotted against the protein concentration. The data points could be fitted with a linear regression where the slope is *k*_cat_/*K*_m_. Each reaction was performed in three replicates**.** The cyclophilin- and FKBP-associated PPIase activities were determined by the extent of inhibition of reaction in the presence of the specific inhibitors CsA and FK506, respectively. The inhibitors were added to the assay mix 30 min before the start of the reaction and incubated at 4 °C. The inhibition constant of CsA for PoxCYP18 and its mutant proteins were determined as a gradient of the line of the best fit from a plot of [CsA]/(1 − *k*/*k*_0_) against *k*_0_/*k* where *k* is the rate constant at any given CsA concentration and *k*_o_ is the rate constant in the absence of CsA^63^. To study the effect of Cu^2+^ on catalytic activity, the purified recombinant proteins (4.4 µM each) were incubated with various concentrations of CuSO_4_ for 30 min at 4 °C and followed by estimation of PPIase activity. The inhibition constants of Cu^2+^ for PoxCYP18 and its mutants were determined as a gradient of the line of the best fit from a plot of [Cu^2+^]/(1 − *k*/*k*_0_) against *k*_0_/*k* where *k* is the rate constant at any given Cu^2+^ concentration and *k*_*o*_ is the rate constant in the absence of Cu^2+^^63^. The slope of the line represents the *K*_i_. Data represent the mean ± S.E of triplicates. To generate oxidized forms the purified recombinant proteins (4.4 µM each) were incubated with 300 mM CuSO_4_ for 30 min at 4 °C. After oxidization, the proteins were treated with EDTA (1 mM) and DTT (10 mM) to study their effect on the Cu^2+^-induced inhibition of PPIase activity. The catalytic activity was estimated by standard PPIase assay as described earlier. The sensitivity of the purified cyclophilins to salt and temperature was studied in the presence of different salt concentrations (up to 3 M NaCl), and by treating the proteins at 45 °C, 50 °C, and 60 °C in a water bath followed by assaying the PPIase activity.

### Role of PoxCYP18 in protecting against heat and salt stress

The *E. coli* BL21(DE3)pLysS cells transformed with non-recombinant pET-28a(+) plasmids (control) or recombinant pET-28a(+) plasmid harboring cDNA for PoxCYP18 and mutated cyclophilins, were grown for 2 h (A_600_ ~ 0.4–0.5) in 20 ml Luria Bertani (LB) broth containing 50 µg ml^−1^ kanamycin. For the imposition of salt stress, 1 ml of each culture was inoculated in 9 ml LB broth in 50 ml tubes containing 50 µg ml^−1^ kanamycin, 0.5 mM IPTG and 500 mM NaCl, and allowed to grow at 37 °C with shaking at 180 rpm for 5 h. For studying the effect of heat stress, 1 ml of culture was inoculated in 9 ml LB broth in 50 ml tubes containing 50 µg ml^−1^ kanamycin, 0.5 mM IPTG, and allowed to grow at 47 °C with shaking at 180 rpm for 5 h. To check the effect of different genes on the survivability of the cells, an equal volume of inoculum (1 ml) from induced *E. coli* cultures was harvested and was serially diluted and spotted on LB agar plates containing kanamycin (50 µg ml^−1^), followed by overnight incubation at 37 °C. All these experiments were performed in two biological replicates.

### Protein structure modeling and preparation

The 3D structure of the native PoxCYP18 protein was modeled using Modeller version 10.1 (https://salilab.org/modeller/)^78^ using crystal structures of cyclophilin protein from Aspergillus fumigatus (PDB id: 2c3b, per cent identity: 78%^79^, and *Piriformospora indica* (PDB id: 4eyv, percent identity: 63%^27^, as templates. Three PoxCYP18 mutant proteins were also modeled using a modeler by replacing cysteine residues with serine at positions 45, 170 and both. ERRAT v2.0^80^, VERIFY3D^81,82^ and PROCHECK^83^ implemented in the SAVES server version 6.0 (https://saves.mbi.ucla.edu/) were used to validate the protein models. The energy of the native and mutant protein models was minimized using the AMBER99SB-ILDN forcefield^84^ and the Steepest Descent method (5000 steps)^85^ implemented in the GROMACS^86^.

### All-atom molecular dynamics (MD) simulations

All-atom MD simulations were performed using Gromacs version 2022.3^87^ with the AMBER99SB-ILDNP forcefield^84^. The solvation of the simulation systems was done using the TIP3P water model, followed by neutralizing the system with ions at a concentration of 150 mM NaCl. The periodic boundary conditions were applied in the form of a dodecahedron box. Energy minimization (in a vacuum as well as in solution) was performed using the steepest descent algorithm, followed by sequential execution of isochoric (NVT) and isobaric (NPT) equilibrations using the Verlet “cutoff” scheme^88^ for van der Waals interactions and the particle-mesh Ewald approach^89^ to take care of long-range electrostatic interactions. Additional steps of isobaric equilibrations were performed to relax the position restraints in a step-wise manner, followed by their complete removal. This equilibrated and unrestrained system was subjected to a 50 ns production MD simulation. Using the V-rescale algorithm, a Verlet cutoff approach was used to refresh the neighbour list every 10 steps while maintaining the temperature at 310 k (τT = 0.1 ps)^90^. The Parrinello-Rahman pressure coupling^91^ was used to keep the pressure at 1 atm (τP = 0.5 ps, compressibility = 4.5 × 10^−5^).

### Statistical analysis

All the experiments were performed in triplicate unless otherwise specified. The data were analyzed by one-way analysis of variance (ANOVA) via Tukey’s multiple comparison test using Graph pad prism 7 software.

### Supplementary Information

Supplementary Information.

## Data Availability

The nucleotide sequence of PoxCYP18 gene used in the current study was deposited in the GenBank data base with accession number of MZ407579.1.
