# Supplementary material for: Characterization and regulation of salt upregulated cyclophilin from a halotolerant strain of Penicillium oxalicum
Source: Sci Rep. 2023 Oct 13;13:17433. doi: 10.1038/s41598-023-44606-5 (PMC10575979; doi:10.1038/s41598-023-44606-5)
Supplement: Supplementary file 1 — Supplementary Information. [file 41598_2023_44606_MOESM1_ESM.pdf]

Title: Characterization and Regulation of Salt Upregulated Cyclophilin from a Halotolerant Strain of *Penicillium oxalicum.*

Authors name:

Mangaljeet Singh[1], Harpreet Singh[2], Kirandeep Kaur[1], Shubhankar[1],

Supreet Singh[1], Amarjeet Kaur[3], Prabhjeet Singh[1]*

[1] Department of Biotechnology, Guru Nanak Dev University, Amritsar, Punjab, India, 143005

[2] Department of Bioinformatics, Hans Raj Mahila Maha Vidyalaya, Jalandhar, Punjab, India 144008

[3] Department of Microbiology, Guru Nanak Dev University, Amritsar, Punjab, India, 143005

*Corresponding author

Email: Mangaljeet Singh- mangaljeetsingh91@gmail.com, Harpreet Singh- harpreetsingh05@gmail.com, Kirandeep Kaur- kiran.91nsr@gmail.com, Shubhankar- shubhankar2653@gmail.com, Supreet Singh- supreetsinghjodhka@gmail.com , Amarjeet Kaur - amarjeet_b@rediffmail.com, Prabhjeet Singh- singhprabhjeet62@gmail.com

Supplementary Table 1: Primers used for amplification of cDNA encoding PoxCYP18 cyclophilin and its mutants from *P. oxalicum.*

Proteins	Primers	Restriction sites	Annealing temperature	Amplicon size (bp)
PoxCYP18	F: CGCGGATCCATGTCCAACACCAAGGCTTTCTTC<br>R: CCGGAATTCTTACATCTCACCACAGTTGACAATG	BamHI<br>EcoRI	58 °C	522
PoxCYP18^{C45S}	F: CGTGAGCTCAGCAAGGCTCCC<br>R: GGGAGCCTTGCTGAGCTCACG	BamHI<br>EcoRI	60 °C	5891
PoxCYP18^{C170S}	F: ATTGTCAACTCTGGTGAGATG<br>R: CATCTCACCAGAGTTGACAAT	BamHI<br>EcoRI	53 °C	5891
PoxCYP18$^{C45S/C170S}$	F: ATTGTCAACTCTGGTGAGATG<br>R: CATCTCACCAGAGTTGACAAT	BamHI<br>EcoRI	53 °C	5891

Supplementary Table 2: Percentage identity and similarity of PoxCYP18 with its orthologous from other fungi. Red color represents lowest and green shows highest percentage similarity.

	1	2	3	4	5	6	7	8	9	% Identity
1. EPS30376.1[*Penicillium oxalicum*]		78	82.8	60.1	59.5	60.7	63	63.2	60.1	
2. CAB44442.1[*Aspergillus_fumigatus*]	87.9		77.6	60.3	58.5	60.2	63	58.6	61	
3. AAD16142.1[*Aspergillus_niger*]	**90.2**	87.4		60.9	62.1	64.4	66.1	62.3	59.8	
4. AAA34336.1[*Candida_albicans*]	72.3	73.7	74.1		76.5	69.8	67.7	71.3	81.5	
5. AAK20862.1[*Cryptococcus_neoformans*]	71.7	70.2	73.6	84		72.8	71.3	67.7	73.5	
6. ACR20103.1[*Moniliophthora_perniciosa*]	71.1	73.7	75.3	80.9	82.7		72.6	66.5	67.3	
7. ACS71332.1[*Piriformospora_indica*]	71.7	73.1	72.4	77.4	82.3	83.5		64.6	65.2	
8. ALM24136.1[*Rhizopus_arrhizus*]	72.3	71.9	73	81.7	78.7	77.4	78		68.3	
9. KAF1910233.1[*Saccharomyces_cerevisiae*]	71.1	74.9	70.7	88.3	82.1	78.4	76.2	81.1		
	% Similarity									

Protein sequences were retrieved from the database, and pairwise percentage sequence identity and similarity were calculated using the Matrix Global Alignment Tool (MatGAT) version 2.02, selecting BLOSUM50 as scoring matrix.

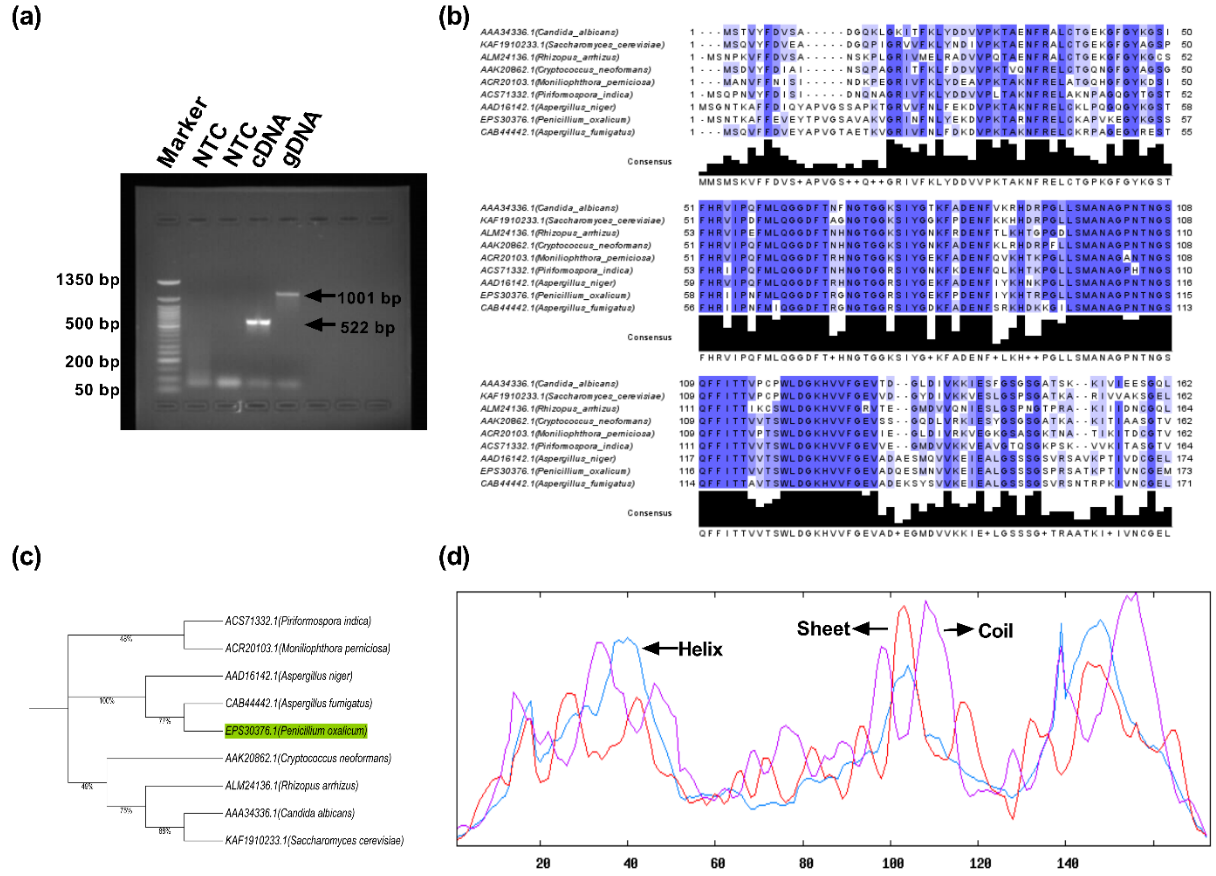

Supplementary Figure S1.

Sequence and phylogenetic analysis. **(a)** Agarose gel (1.6%) electrophoresis of amplicons obtained after a polymerase chain reaction of cDNA and genomic DNA (gDNA) with gene-specific primers of *PoxCYP18* (NTC: no-template control). **(b)** Multiple sequence alignment analysis of PoxCYP18 with other fungal cyclophilins was performed by using MUSCLE algorithm in Jalview software (v2.11.1.3) (http://www.jalview.org/). Identical residues are shown in blue color. **(c)** Phylogenetic analysis (http:// www.megasoftware.ne) of the amino acid sequences of PoxCYP18 with homologous protein sequences of previously known fungal cyclophilins. The unrooted tree was generated using the neighbor-joining method in MEGA X (v10.1.7) software (http:// www.megasoftware.ne) and the numbers on the branches represent the bootstrap values for 1000 replicates. **(d)** Distribution of secondary structure elements in PoxCYP18. Red, purple and blue colors represent the sheet, coil and helix respectively. The x-axis shows the number of amino acids.

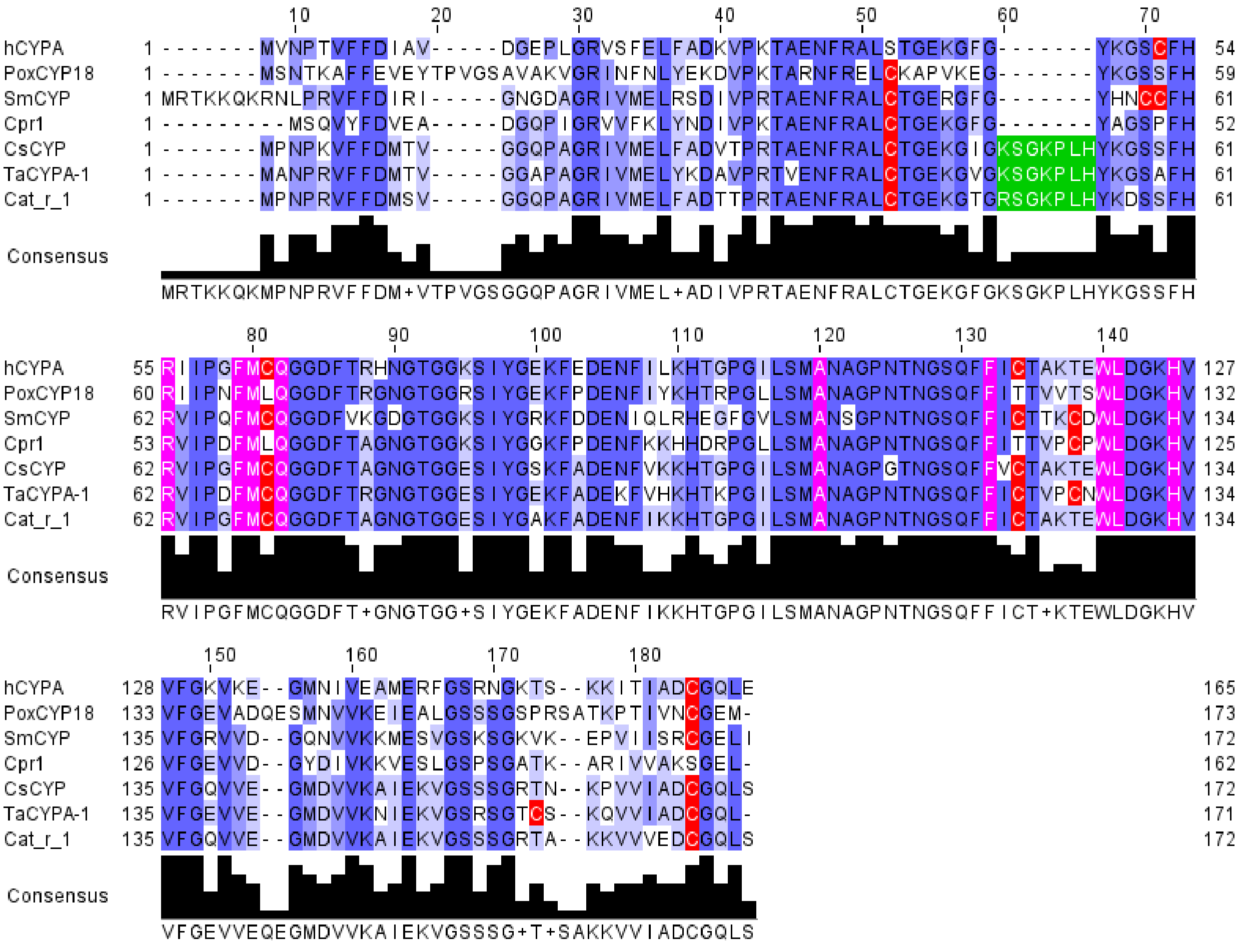

Supplementary Figure S2.

Multiple sequence alignment of PoxCYP18 with cyclophilins from human (hCYPA), *Schistosoma mansoni* (SmCYP), *Saccharomyces cerevisiae* (Cpr1), *Citrus sinensis* (CsCYP), wheat (TaCYPA-1), and *Catharanthus roseus* (Cat_r_1) performed by using MUSCLE algorithm in Jalview software (v2.11.1.3) (http://www.jalview.org/). Residues reported to be important for PPIase activity and cyclosporin A interaction are highlighted in Pink. Residues constituting the divergent loop are marked in green, while the cysteine residues are indicated in red

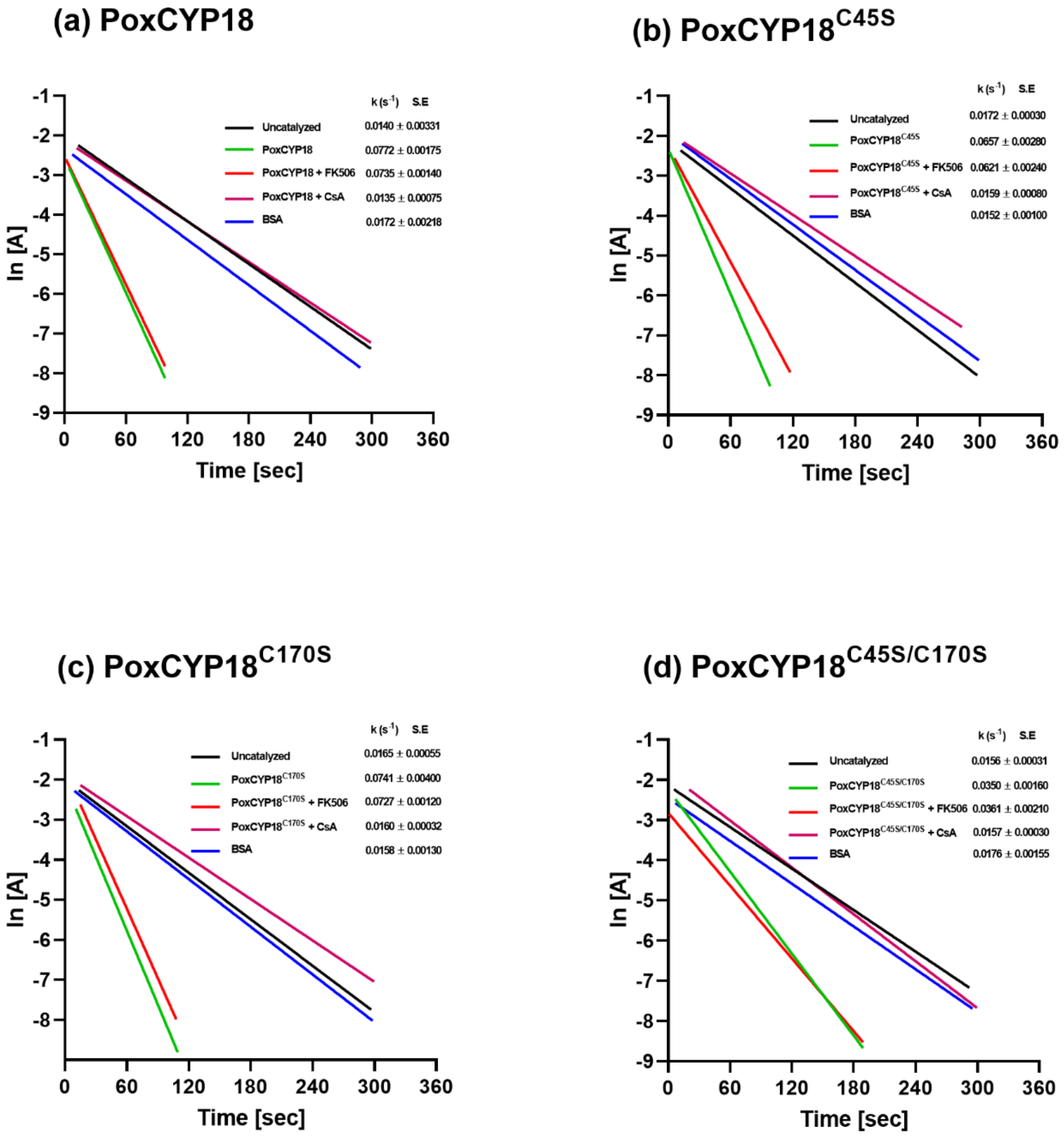

Supplementary Figure S3.

Plots of natural logarithm of absorbance [A] vs time (sec) depicts the first order rate of reaction for **(a)** PoxCYP18 and mutants PoxCYP18^{C45S} **(b)**, PoxCYP18^{C170S} **(c)** and PoxCYP18$^{C45S/C170S}$ **(d)**. Data represent the mean ± S.E of three replicates.

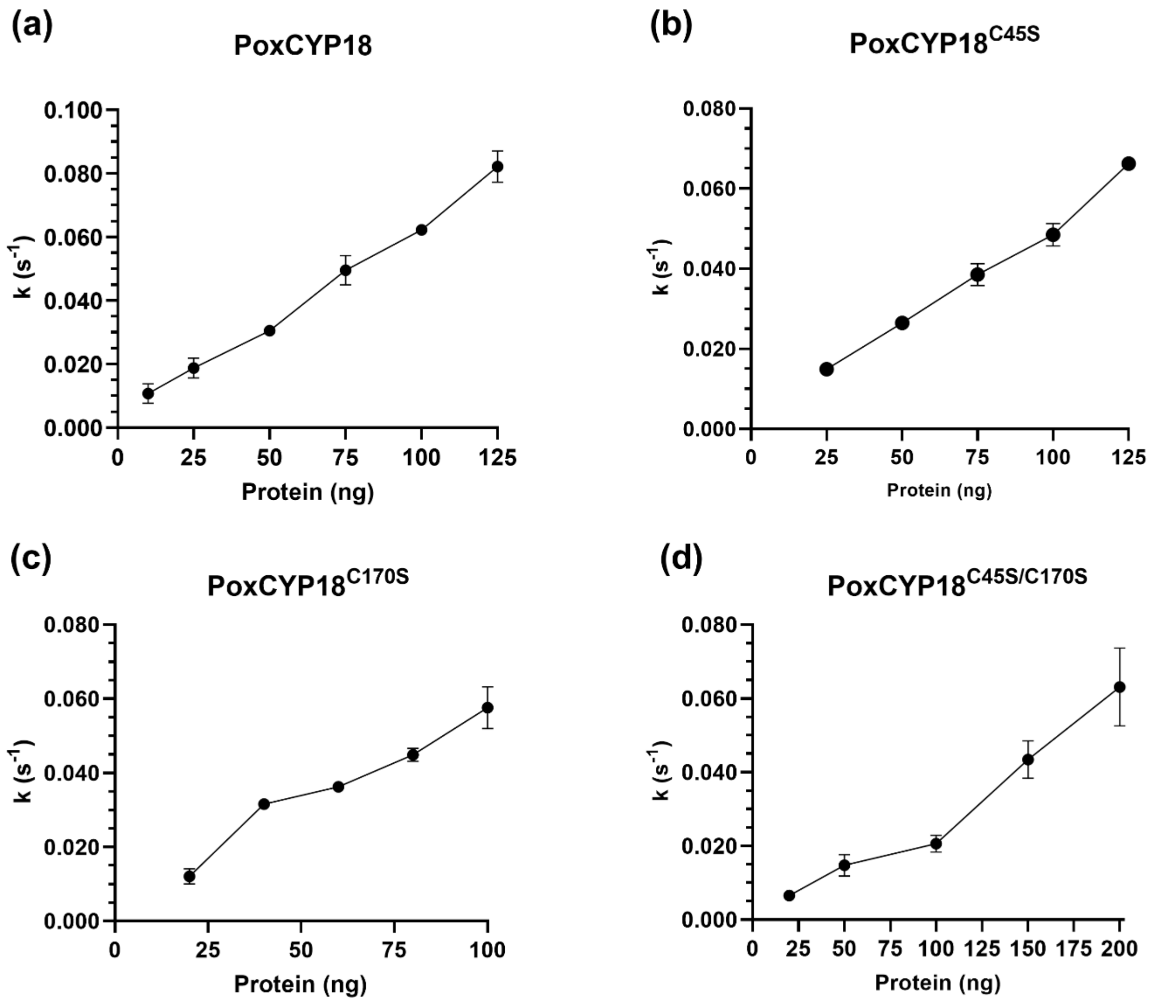

Supplementary Figure S4.

Effect of different amounts of purified PoxCYP18 **(a)** and mutant proteins; PoxCYP18^{C45S} **(b)**, PoxCYP18^{C170S} **(c)** and PoxCYP18$^{C45S/C170S}$ **(d)** on the rate constant. Data represent the mean ± S.E of three replicates.

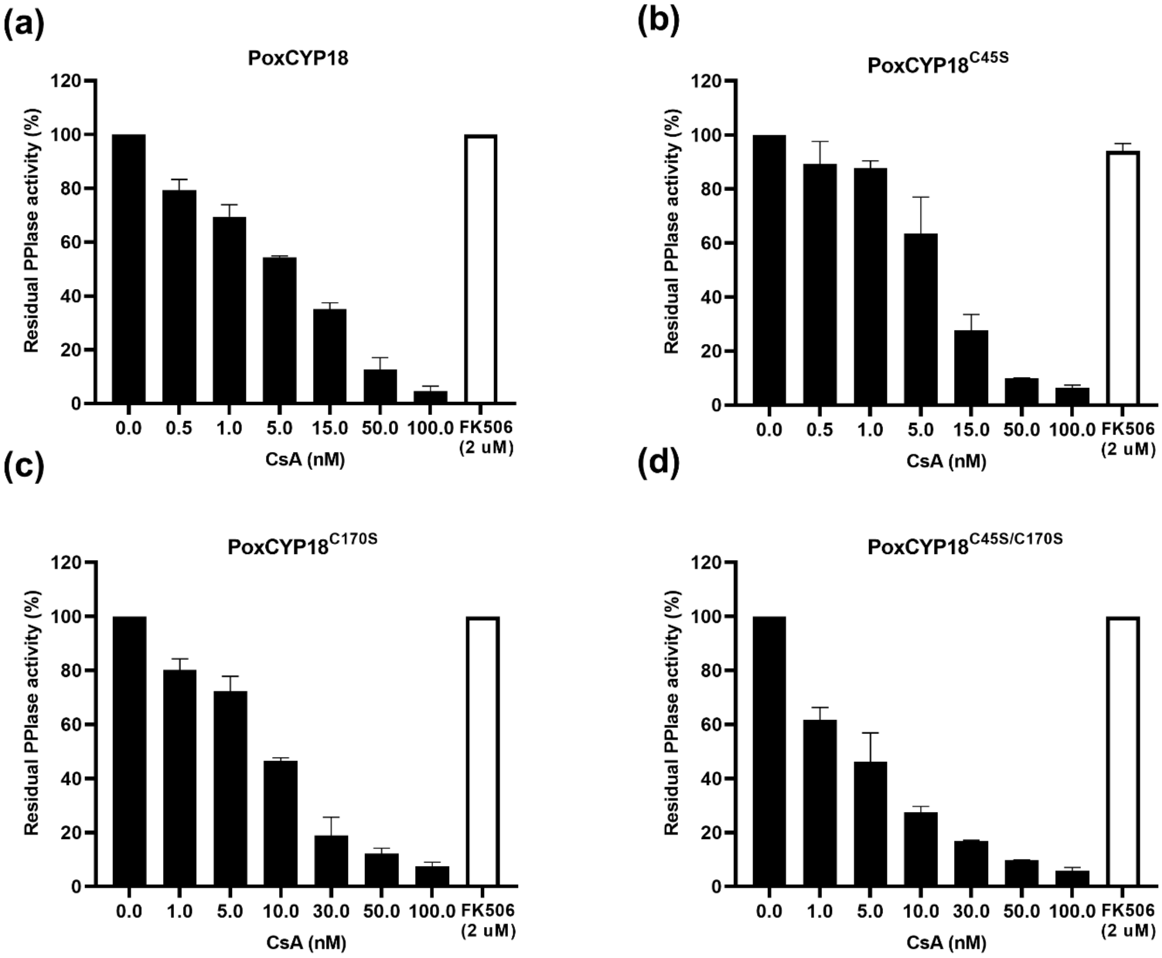

Supplementary Figure S5.

Effect of cyclophilin inhibitor, cyclosporin A (CsA), and FK506-binding protein inhibitor, FK506, on the PPIase activity of native PoxCYP18 **(a)** and mutants PoxCYP18^{C45S} **(b)**, PoxCYP18^{C170S} **(c)** and PoxCYP18$^{C45S/C170S}$ **(d)**. The purified proteins were incubated with CsA and FK506 before carrying out PPIase assay. Data represent the mean ± S.E of three replicates.

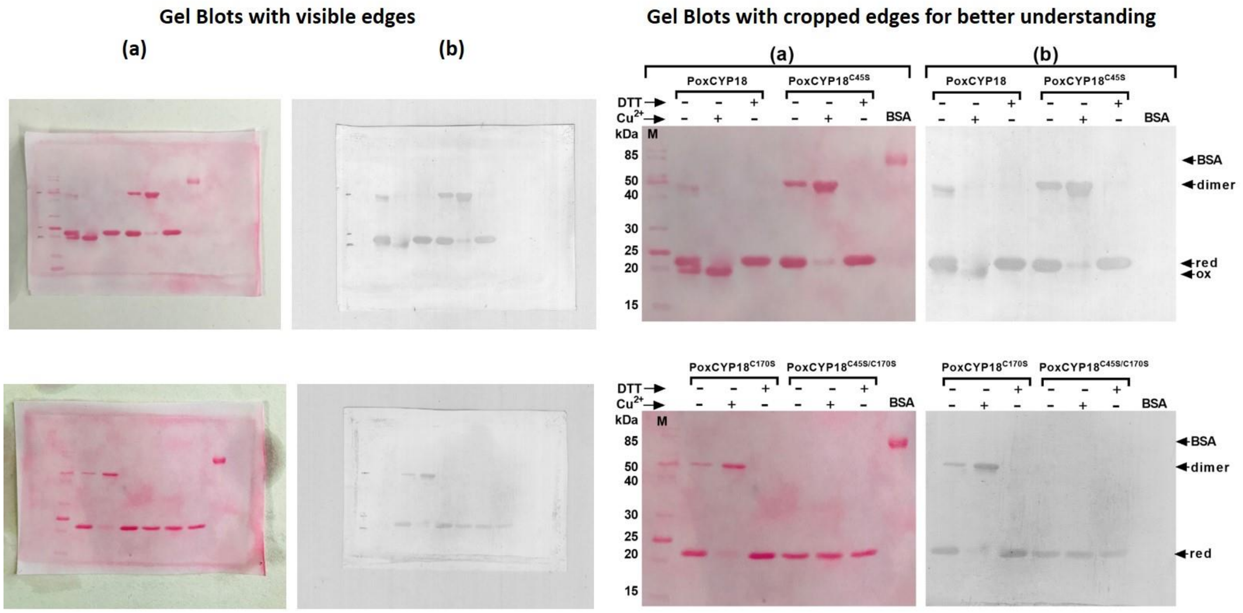

Supplementary Figure S6.

Confirmation of the purified recombinant proteins by immunoblotting with the anti-His antibodies. Untreated (absence of DTT and Cu^{2+}), oxidized (10 mM Cu^{2+}) and reduced (50 mM DTT) samples of PoxCYP18 and its mutants were resolved by SDS-PAGE (12%), followed by Ponceau staining **(a)** and western blotting **(b)** with anti-His antibody. BSA was used as the negative control.

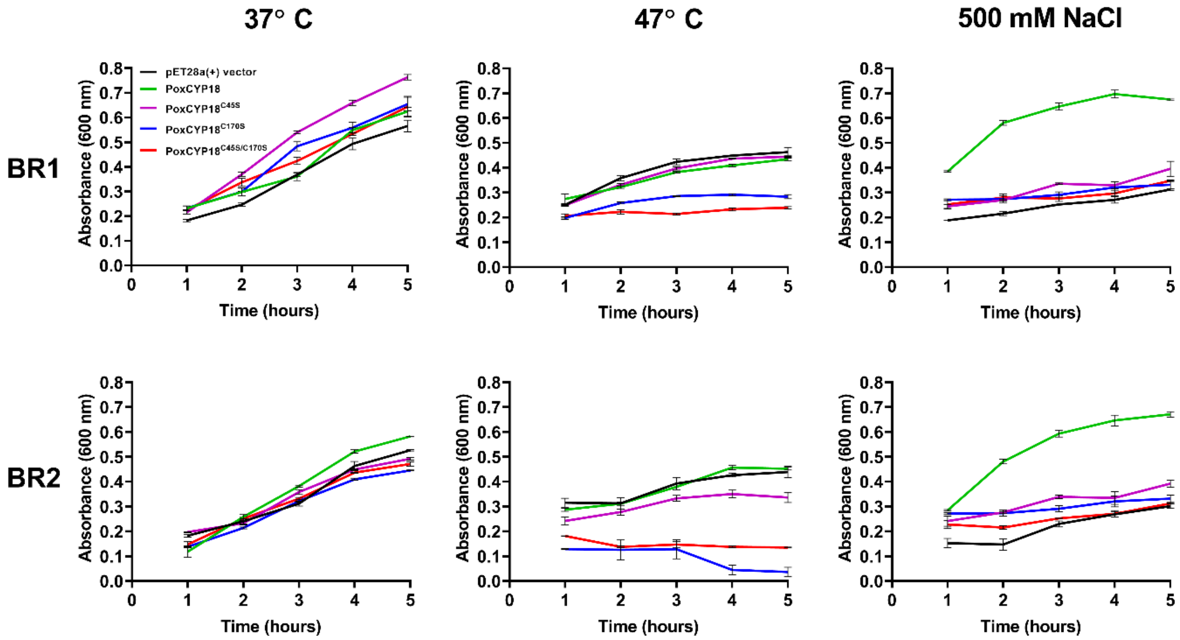

Supplementary Figure S7: Stress tolerance assay of *E. coli* BL21(DE3)pLysS cells overexpressing PoxCYP18 and its mutants, PoxCYP18^{C45S}, PoxCYP18^{C170S} and PoxCYP18$^{C45S/C170S}$, under salt (500 mM) and heat (47°C) stress conditions. The growth of the *E. coli* BL21(DE3)pLysS cells transformed with *PoxCYP18* and its mutants was compared by monitoring absorbance at 600 nm relative to the controls transformed with non-recombinant pET-28a(+). Data represent the mean ± S.E of three technical replicates from each of the two biological replicates, BR1 and BR2.

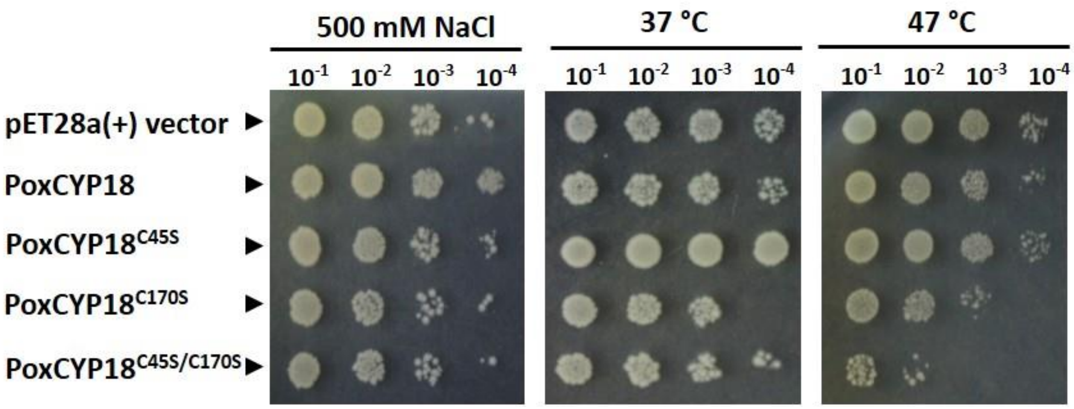

Supplementary Figure S8 (Biological Replicate 2): Effect of PoxCYP18 and its mutants, PoxCYP18^{C45S}, PoxCYP18^{C170S} and PoxCYP18$^{C45S/C170S}$, on salt (500 mM) and heat (47°C) stress tolerance of *E. coli* BL21(DE3)pLysS cells. The growth of the *E. coli* BL21(DE3)pLysS cells transformed with *PoxCYP18* and its mutants on Luria-Bertani media was compared relative to the controls transformed with non-recombinant pET-28a(+).

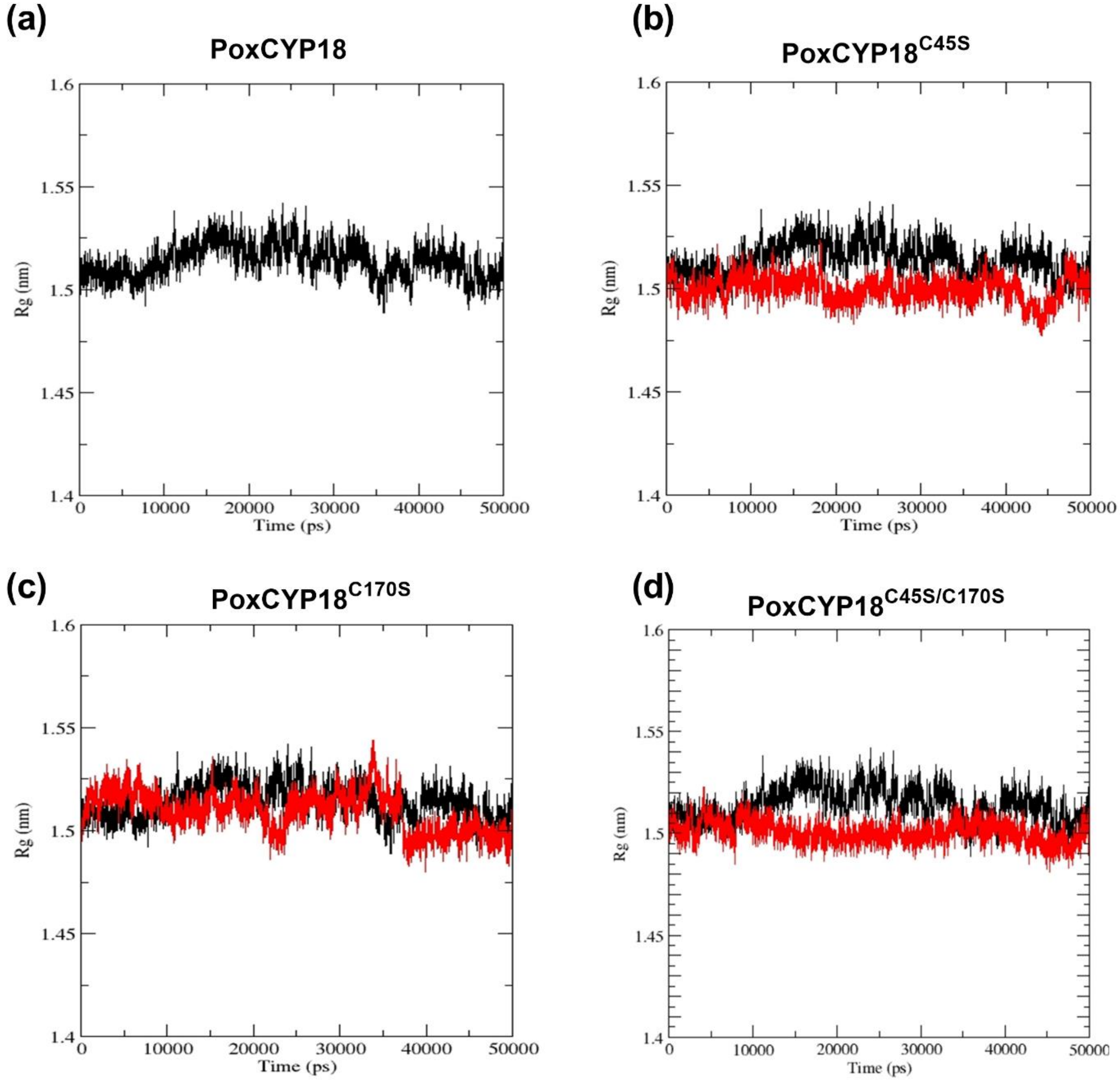

Supplementary Figure S9.

Radius of gyration (Rog) variation in PoxCYP18 (black) and respective mutants (red). (**a**) Rog variation in PoxCYP18. (**b)** Rog variation in PoxCYP18^{C45S} relative to PoxCYP18. **(c)** Rog variation in PoxCYP18^{C170S} relative to PoxCYP18. (**d)** Rog variation in PoxCYP18$^{C45S/C170S}$ relative to PoxCYP18.
